# Synthesis of large-area multilayer hexagonal boron nitride for high material performance

**DOI:** 10.1038/ncomms9662

**Published:** 2015-10-28

**Authors:** Soo Min Kim, Allen Hsu, Min Ho Park, Sang Hoon Chae, Seok Joon Yun, Joo Song Lee, Dae-Hyun Cho, Wenjing Fang, Changgu Lee, Tomás Palacios, Mildred Dresselhaus, Ki Kang Kim, Young Hee Lee, Jing Kong

**Affiliations:** 1 Institute of Advanced Composite Materials, Korea Institute of Science and Technology (KIST), San101 Eunha-Ri, Bongdong-Eup, Wanju-Gun, Jeollabuk-Do 565-902, Korea; 2 Department of Electrical Engineering and Computer Sciences, Massachusetts Institute of Technology, Cambridge, Massachusetts 02139, USA; 3 School of Advanced Materials Science and Engineering, Sungkyunkwan University, Suwon 440-746, Korea; 4 Center for Integrated Nanostructure Physics (CINAP), Institute for Basic Science (IBS), Sungkyunkwan University, Suwon 440-746, Korea; 5 Department of Energy Science, Department of Physics, Sungkyunkwan University, Suwon 440-746, Korea; 6 Department of Mechanical and Aerospace Engineering, The Ohio State University, Columbus, Ohio 43210-1142, USA; 7 School of Mechanical Engineering, Sungkyunkwan University, Suwon 440-746, Korea; 8 SKKU Advanced Institute of Nanotechnology, Sungkyunkwan University, Suwon 440-746, Korea; 9 Department of Physics, Massachusetts Institute of Technology, Cambridge, Massachusetts 02139, USA; 10 Department of Energy and Materials Engineering, Dongguk University-Seoul, Seoul 100-715, Korea

## Abstract

Although hexagonal boron nitride (h-BN) is a good candidate for gate-insulating materials by minimizing interaction from substrate, further applications to electronic devices with available two-dimensional semiconductors continue to be limited by flake size. While monolayer h-BN has been synthesized on Pt and Cu foil using chemical vapour deposition (CVD), multilayer h-BN is still absent. Here we use Fe foil and synthesize large-area multilayer h-BN film by CVD with a borazine precursor. These films reveal strong cathodoluminescence and high mechanical strength (Young's modulus: 1.16±0.1 TPa), reminiscent of formation of high-quality h-BN. The CVD-grown graphene on multilayer h-BN film yields a high carrier mobility of ∼24,000 cm^2^ V^−1^ s^−1^ at room temperature, higher than that (∼13,000 ^2^ V^−1^ s^−1^) with exfoliated h-BN. By placing additional h-BN on a SiO_2_/Si substrate for a MoS_2_ (WSe_2_) field-effect transistor, the doping effect from gate oxide is minimized and furthermore the mobility is improved by four (150) times.

## Abstract (web summary)

Multilayer h-BN films are highly desired for various applications in 2D nanoelectronics. Here, the authors demonstrate the synthesis of large-area and high-quality multi-layer h-BN films on Fe foil with high 2D material performance.

## Graphical abstract

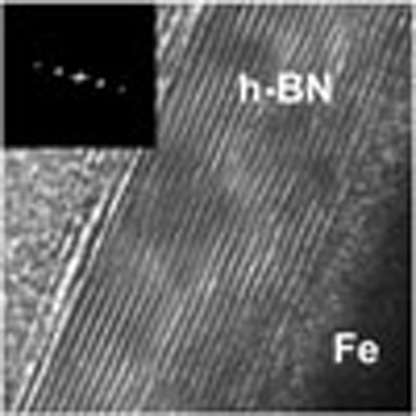

In addition to metallic graphene and semiconducting transition metal dichalcogenides, hexagonal boron nitride (h-BN) is an insulating two-dimensional (2D) material, in which all these materials serve as primary components for soft electronics with high flexibility and transmittance^1^,^2^. Since all the atoms in 2D layer are exposed to the surface, the related physical and chemical properties are strongly influenced by adjacent materials and sometimes surface corrugation^3^,^4^. Therefore, a special care is required to deal with atomically thin-layered materials. h-BN has several unique physical and chemical properties of its own, which has potential applications as a dry-lubricant^5^, passivation layer^6^ and deep ultraviolet emitter^7^,^8^,^9^. The h-BN layer has been recently demonstrated to be an ideal substrate for 2D materials due to its atomic flatness, large optical bandgap, superb mechanical strength, absence of dangling bonds and low dielectric screening^10^,^11^,^12^,^13^,^14^,^15^,^16^.

So far, tremendous efforts have been provided to large-area synthesis of 2D materials. For example, a meter-scale polycrystalline and centimetre-scale monocrystalline monolayer graphene have been synthesized on copper foil^17^,^18^. A centimeter-scale monolayer transition metal dichalcogenide (TMdC) has been synthesized on an insulator or Au substrate^19^,^20^,^21^,^22^. Several methods including ultrahigh vacuum chemical vapour deposition (CVD) on various single-crystalline transition metals including Ni (111)^23^,^24^, Cu (111)^25^,^26^, Pt (111)^27^ and Ag (111)^28^ have been reported to achieve large-area monolayer h-BN. More recently, more scalable synthesis techniques on polycrystalline Cu and Pt foils were used to obtain monolayer h-BN by CVD^11^,^29^,^30^,^31^. Monolayer h-BN due to its ultrathin nature is very useful for a variety of applications, such as growth templates^32^, tunnelling barriers^33^,^34^ and atomic membranes^35^. Yet, multilayer h-BN is in general highly desired^3^,^36^,^37^,^38^ for numerous real device applications, such as dielectric layers, atomically flat and dangling bond-free substrates or mechanical membranes.

For the synthesis of multilayer h-BN, liquid–metal method^39^, solid-phase epitaxy growth^10^,^40^, direct growth in CVD^13^ and co-segregation method^41^ have been reported. The liquid–metal method so far produces the highest crystallinity but the bulk crystals are produced, resulting in isolated small-size flakes of h-BN after exfoliation^3^. Solid-phase epitaxial growth involves the low-temperature deposition of precursors followed by a post-annealing process, which provides large-area h-BN film with a controllable thickness; however, expensive single-crystalline substrates such as Ni (111) or Ru (0001) are required^10^,^40^. Other methods such as the direct growth with atmospheric pressure CVD on copper foil^13^ or the diffusion and co-segregation of boron and nitrogen atoms from a Fe–Ni alloy^41^ leaves poor crystallinity of h-BN, making it difficult to serve as a substrate for 2D materials. Therefore, improving the crystallinity of large-area h-BN films on a cheap substrate is still required for electronic device applications.

Here we report large-area h-BN films grown by CVD using Fe foil. The thickness of the h-BN (5–15 nm) is controlled by the cooling rate, that is, the segregated boron and nitrogen atoms that are precipitated in Fe substrate at high temperature. X-ray diffraction measurements exclusively revealed the (0002), (0004) and (0006) peaks, indicating that the layers are well aligned perpendicular to the *c* axis. A Young's modulus of 1.16 TPa for a 15-nm-thick h-BN is measured by nanoindentation using atomic force microscope (AFM) tip. All these values are in good agreement with theoretical predictions, indicating the high crystalline quality of the grown multilayer h-BN films. Field-effect transistors (FETs) with CVD-grown monolayer graphene, monolayer MoS_2_ and monolayer WSe_2_ are fabricated on the grown multilayer h-BN substrates, achieving carrier mobilities as high as ∼24,000, 40 and∼9 cm^2^ V^−1^ s^−1^ at room temperature, respectively. The reported graphene mobility is the highest value among those of the previous reports with CVD-grown graphene samples on CVD-grown h-BN substrates.

## Results

### Synthesis and materials characterization of multilayer h-BN

**Figure 1:**
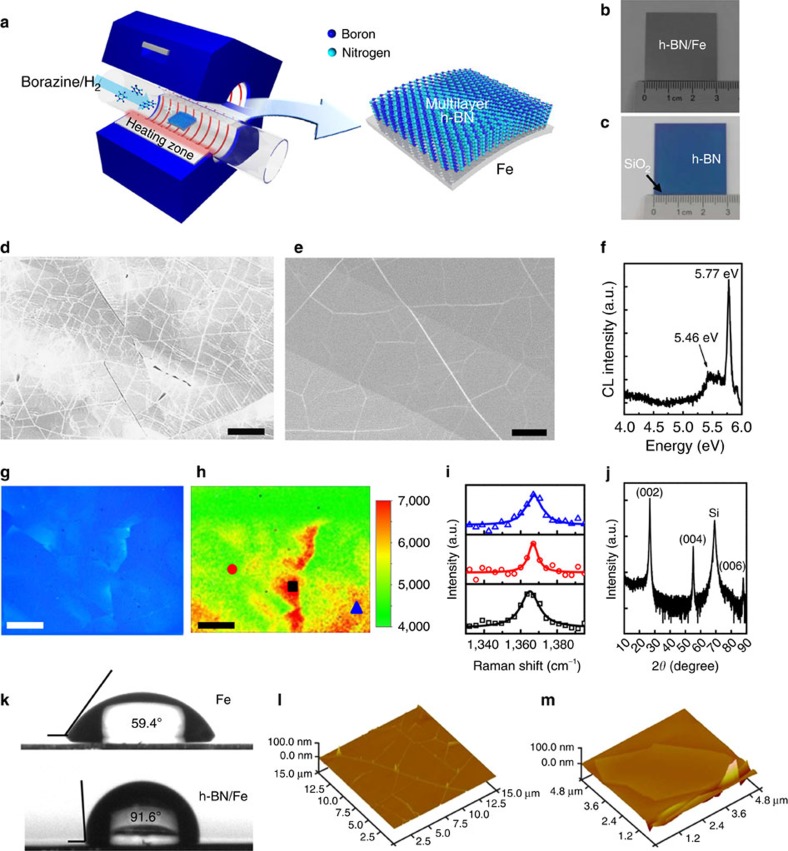
Synthesis of multilayer h-BN film. (**a**) Schematic diagram of the chemical vapour deposition approach for h-BN synthesis. Borazine is used as a precursor. A multilayer h-BN film is grown on a Fe foil in a quarts tube. (**b**,**c**) Photographs of as-grown h-BN film on a Fe foil and the transferred h-BN film onto a SiO_2_/Si substrate. (**d**,**e**) SEM images of an h-BN film on a Fe foil. (**f**) Cathodoluminescence spectra of multilayer h-BN film. (**g**) Optical image of multilayer h-BN film. (**h**) Raman mapping image of the E_2g_ peak near 1,366 cm^−1^ corresponding to the area of **g**. (**i**) Raman spectra of each spot for the corresponding blue triangle, red circle and black square in **h**. (**j**) X-ray diffraction pattern of multilayer h-BN film on a SiO_2_/Si substrate. (**k**) Contact angles of bare Fe (top) and as-grown h-BN on a Fe foil (bottom). (**l**,**m**) AFM images of multilayer h-BN film (**l**) and exfoliated h-BN flake (**m**) on a SiO_2_/Si substrate. The surface roughness of the multilayer h-BN film, except for the wrinkled region and exfoliated h-BN film, is found to be ∼0.2 nm indicating that these CVD-grown multilayer h-BN films are highly flat. Scale bars, 10 μm (**d**); 5 μm (**e**); 20 μm (**g**,**h**).

To synthesize multilayer h-BN on Fe foil, a single-zone CVD furnace is equipped with a bubbling system for liquid borazine, as shown in Fig. 1a^29^. Typically, the growth was performed at a temperature of 1,100 °C for 30 min, followed by various cooling rates (see the Methods section for details). Figure 1b,c shows optical photographs of two 3 × 3-cm^2^ area h-BN samples of as-synthesized on Fe and after transfer onto SiO_2_/Si, respectively. The size of h-BN is only limited by the CVD chamber size. The scanning electron microscopy (SEM) images in Fig. 1d,e are taken of the as-grown h-BN on Fe foil; regions of uniform thickness have typical lateral dimensions of tens of micrometre and without any deposited particles being observed. Several wrinkles can be also seen due to the coefficient of thermal expansion mismatch between the film and the underlying substrate, similar to graphene grown on metallic substrates^42^. Once the h-BN film is delaminated from Fe substrate through a bubble transfer process^29^, the Fe substrate can be reused for future growths. Even after 10 synthesis and transfers of h-BN on the reused Fe substrate, the morphology and quality of the growth results were confirmed to be almost identical (Supplementary Fig. 1).

While reports from the literature have shown both surface-mediated growth, as well as bulk precipitation of h-BN experimentally, the synthesis of h-BN on Fe foils is strongly dependent on the cooling rate of the sample. This strongly suggests that the precipitation plays an important (or even dominant) role in the growth. The h-BN was grown for 30 min with a borazine precursor at 1,100 °C and then fast (30 °C min^−1^) and slow (5 °C min^−1^) cooled until 700 °C without supplying additional borazine (Supplementary Fig. 2). Supplementary Figure 2b–f shows the SEM images of h-BN on Fe foil for fast (Supplementary Fig. 2b,c) and slow (Supplementary Fig. 2e,f) cooling, respectively. In the case of fast cooling (Supplementary Fig. 2b,c), a relatively thin h-BN film (an average thickness of 11.9 nm with a s.d. of 3.3 nm) with triangular islands covered the Fe surface (Supplementary Figs 3 and 4). The wrinkles of the background h-BN film are all merged together, indicating that the h-BN film is continuous; on the other hand, slow cooling results in a thicker h-BN film (an average thickness of 17.8 nm with a s.d. of 6.1 nm over the whole area (Supplementary Fig. 2e,f). Moreover, the grain size of h-BN film was roughly estimated using polarizing optical microscopy assisted by the nematic 5CB liquid crystal^29^. The grain size of h-BN for fast- and slow-cooling samples was obtained to be at least 40,000 and 160,000 μm^2^, respectively (Supplementary Fig. 5). Therefore, the h-BN film might be grown via both surface-mediated growth^29^ and precipitation^39^. While it is impossible to exclude the possibility that the initial h-BN film could be a surface-mediated growth (that is, Frank-van Der Merwe model), the effect of the slower cooling rate suggests that boron and nitrogen are segregated from bulk iron to form thick layers of h-BN on the surface. At these growth temperatures, boron atoms can easily react with iron to form FeB_*x*_ (*x*=1 and 2), leading to boron atoms dissolving into iron bulk through a reaction–diffusion mechanism^41^,^43^; in addition, there is also a finite solubility of N in Fe (∼8 at. %) at 1,000 °C (ref. 44).

Cathodoluminescence (CL) and Raman spectroscopy were used to understand the optical properties of the h-BN film. Figure 1f shows the CL spectrum of the grown h-BN after transfer. The peaks at 5.77 and 5.46 eV assigned to the exciton of h-BN (called the S-line) and the excitons trapped to structural defects (called the D-line) were clearly observed^45^,^46^,^47^. Such CL spectra have only been reported for the highest quality of bulk h-BN grown by the liquid–metal method^39^. The integrated intensity ratio of D/S is small (0.275), which is comparable to that of single-crystalline h-BN^47^. This result implies that the quality of our h-BN film is similar to that of the single-crystalline bulk h-BN. Figure 1g,h shows the optical image of a 100 × 100-μm^2^ region and the corresponding Raman mapping image of the E_2g_ peak (near 1,366 cm^−1^)^48^. The Raman intensity changes slightly at different locations, corresponding to the thickness variation of the h-BN regions, but the E_2g_ Raman peak is observed in all regions, indicating that the h-BN film is continuous over the whole area. Figure 1i shows the Raman spectra of the three different locations (indicated by a triangle, square and circle in Fig. 1h, respectively). Only the E_2g_ Raman peak is observed, which is a characteristic of a h-BN structure, as opposed to cubic BN that gives rise to Raman modes at 1,056 (TO phonon) and 1,306 cm^−1^ (LO phonon)^49^. To estimate the stacking order and crystallinity, the multilayer h-BN films on SiO_2_/Si substrate are further characterized by X-ray diffraction. The X-ray diffraction patterns in Fig. 1j are in good agreement with the previous works^50^,^51^. Interestingly, only (0002), (0004) and (0006) peaks are observed, indicating that these multilayers were well aligned with the *c* axis perpendicular to the Fe substrate. Figure 1k shows the contact angle measurement for the h-BN film grown on Fe compared with the bare Fe substrate. The contact angle is increased by >30°, suggesting that these h-BN films can be used as hydrophobic surface coatings.

Another important quantity for various device applications is the surface flatness for these h-BN films. Figure 1l,m shows the AFM images of CVD-grown h-BN film and an exfoliated single-crystalline h-BN flake, respectively. The surface morphology of the two is quite comparable; roughness values of ∼0.2 nm are obtained for both samples, confirming the high quality of the CVD-grown samples. Additional AFM images of the as-grown h-BN on Fe and transferred h-BN on SiO_2_ (top view) are shown in Supplementary Figs 6 and 7. The average terrace size of h-BN film was roughly obtained to be 10 × 10 μm^2^ or larger from AFM morphology analysis (Supplementary Fig. 6).

### Characterization of structural quality of multilayer h-BN

**Figure 2:**
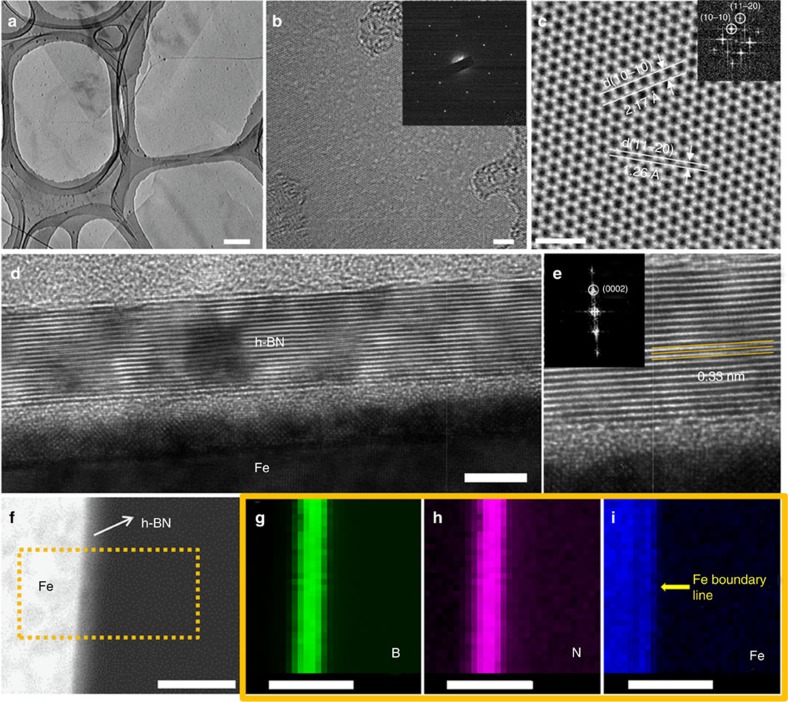
Atomic structure of multilayer h-BN film. (**a**,**b**) Low- and high-magnification TEM images of a multilayer h-BN film. A hexagonal diffraction pattern, as shown in the inset of **b**, indicates that the h-BN film is well stacked with an AA′ stacking order. (**c**) High-angle annular dark-field STEM image of a multilayer h-BN film. The inset shows the fast Fourier transform (FFT) image corresponding to **c**. The (10–10) and (11–20) lattice planes are identified. The d-spacing of the (10–10) and (11–20) lattice planes are confirmed to be 2.17 and 1.26 Å, respectively. (**d**,**e**) Cross-sectional TEM images of an as-grown multilayer h-BN film on a Fe foil. The (0002) lattice plane with a d-spacing of 0.33 nm in **e** is identified from the FFT image. (**f**) Low-magnification TEM image at the interface region between the Fe and multilayer h-BN. (**g**–**i**) EELS mapping images of boron, nitrogen and Fe atoms, respectively, corresponding to the region of the dotted box in **f**. Scale bars, 5 μm (**a**); 2 nm (**b**,**c**); 5 nm (**d**); 20 nm (**f**–**i**).

To estimate the structural quality of h-BN film, the samples were characterized by transmission electron microscopy (TEM). Figure 2a shows a low-magnification image of the h-BN film suspended on the TEM grid. Compared with previous h-BN films synthesized by Ni or Cu, these films are highly continuous^10^,^11^. The high-magnification image in Fig. 2b exhibits the lattice fringes of h-BN. The selective area diffraction pattern image shown in the inset of Fig. 2b displays a single hexagonal pattern, indicating that these multilayer h-BN samples are well stacked with an AA′ stacking order^50^. Figure 2c shows an enlarged image of Fig. 2b to highlight the clearly observed hexagonal atomic arrangement of boron and nitrogen atoms. The d-spacings of (10–10) and (11–20) planes are obtained to be 2.17 and 1.26 Å, respectively, which are in good agreement with theoretical values^52^. Figure 2d displays the cross-sectional TEM image of h-BN grown on the Fe foil. The regions between h-BN and Fe are clearly separated and the h-BN film is highly continuous (Supplementary Fig. 8 for different cross-sectional TEM images). Figure 2e shows a higher magnification of the cross-sectional TEM image. An interlayer distance of 0.33 nm high-magnification TEM images of a multi-layer X-ray diffraction characterization of high-quality bulk h-BN^51^. The fast Fourier transform image in the inset of Fig. 2e further confirms the orientation of the (0002) lattice planes, corroborating the X-ray diffraction results. Electron energy loss spectroscopy (EELS) mapping was carried out to understand the composition of Fe, boron and nitrogen in the h-BN, as well as the stoichiometry of boron and nitrogen. Figure 2f is the low-magnification TEM image and Fig. 2g–i displays the corresponding EELS mapping images for B, N and Fe, respectively. The boron and nitrogen atoms are experimentally found only on the top of the iron surface to form the h-BN film. It was also confirmed that the stoichiometry of boron and nitrogen is 1:1 through the EELS measurement, as well as through X-ray photoemission spectroscopy (Supplementary Figs 9 and 10).

### Mechanical strength of multilayer h-BN film

**Figure 3:**
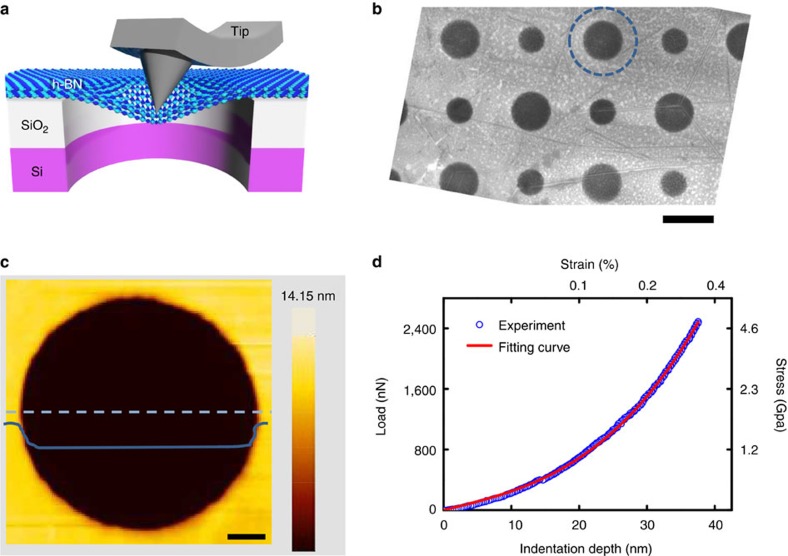
Mechanical strength of multilayer h-BN film. (**a**) Schematic diagram of the measurements by AFM nanoindentation. (**b**) SEM image of a multilayer h-BN film onto a SiO_2_/Si substrate with patterned circular holes having diameters of 1.5 and 1 nm with a depth of 500 nm. Thickness of the tested h-BN film is ∼15 nm. (**c**) AFM image of the suspended multilayer h-BN film. The solid line indicates the height profile along the dotted line. The h-BN membrane has already sagged into the hole by 15 nm before indentation. (**d**) Mechanical response of the h-BN film by nanoindentation. By fitting the curve by the technique in ref. 53, an average elastic modulus of 18,000 Nm^−1^ is obtained, which corresponds to a Young's modulus of 1.16 TPa. Scale bars, 1 μm (**b**); 0.2 μm (**c**).

An AFM was used to measure the elastic modulus of the h-BN film through a nanoindentation technique^53^. Figure 3a shows a schematic diagram of the measurement system. Before performing nanoindentation tests, the h-BN film was transferred onto a SiO_2_ substrate with patterned circular holes (diameters 1.5 and 1 μm, and depth 500 nm). The thickness of the tested h-BN film was ∼15 nm (Supplementary Fig. 11). Figure 3b shows the h-BN film placed above circular holes to form an array of suspended membranes maintaining high flatness. It was confirmed that the membrane was tightly clamped to the sidewalls of the circular patterns by examining the tapping mode image (Fig. 3c). The solid blue line in Fig. 3c is a height profile along the dashed line, indicating that the h-BN membrane had already sagged into the hole by 15 nmbefore indentation. By indentation the centre of the suspended membranes, the mechanical deformation of the h-BN film is obtained (Fig. 3d), which results in a measured average elastic modulus of 18,000 Nm^−1^ corresponding to a Young's modulus of 1.16±0.1 TPa (ref. 53). The measured value agrees within experimental error with the theoretically calculated Young's modulus of single-layer h-BN (0.995 TPa for biaxial strain)^54^. Even for a given load of 2,400 Nm, corresponding to a strain and stress of 0.35% and 37 GPa, respectively, the film was still intact demonstrating the remarkably high mechanical strength of these multilayer h-BN films.

### Dielectric properties of multilayer h-BN film

**Figure 4:**
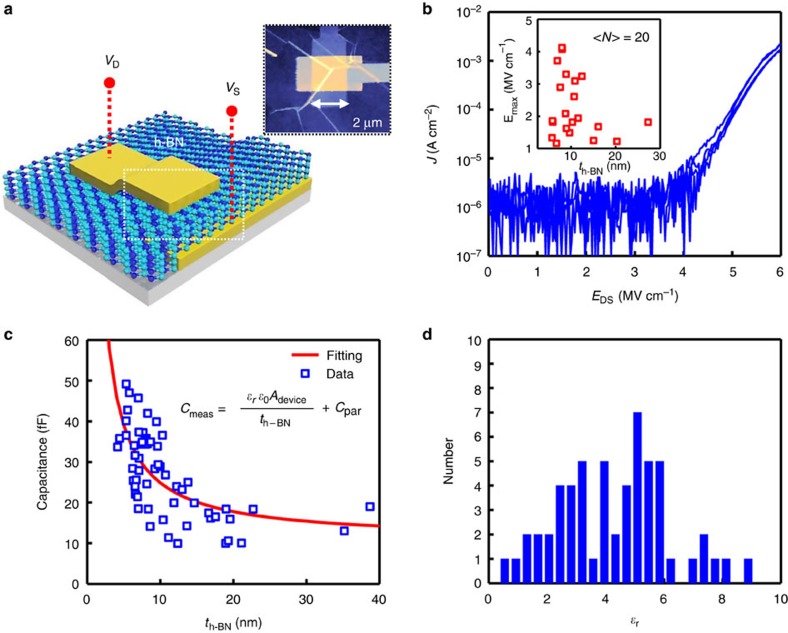
Dielectric properties of multilayer h-BN and graphene on an h-BN substrate. (**a**) Schematic diagram of a metal (Pt)–insulator (h-BN)–metal (Pd) capacitor structure. The inset shows an AFM image of a real device. (**b**) Representative current density (*J*) versus *E*_DS_ applied electric field for an h-BN film of 10-nm thick. Breakdown current at ∼10^−5^ A cm^−2^ is defined, which is just above the noise floor of the measurement system. The inset displays the breakdown electric field for devices with various thicknesses. (**c**) Capacitance as a function of the h-BN thickness (*t*_h-BN_). The parasitic capacitance (*C*_par_) was extracted by fitting the data point with equation (1). A typical *C*_par_ of ∼15 fF in relatively thick devices was extracted. (**d**) Histogram of the number versus dielectric constant of h-BN

To evaluate the electrical properties of these h-BN films as a dielectric material, metal–insulator–metal structures were fabricated to measure the breakdown electric field and relative dielectric constant (*ɛ*_r_) of the h-BN^3^,^37^. Figure 4a shows the schematic of an metal–insulator–metal device (see the Methods section for details). All devices (*N*=20) measured for the breakdown electric field consisted of a 1 × 1 μm^2^ overlap of electrodes. The inset in Fig. 4a shows AFM image of a typical device. Figure 4b shows a representative current density (*J*) versus applied electric field (*E*_SD_) for an h-BN film with a thickness of ∼10 nm. A breakdown current density of ∼10^−5^ A cm^−2^ is defined, which is just above the noise floor of the measurement system. The electric field was computed by measuring the thickness of the h-BN for each device using AFM. The inset in Fig. 4b shows the breakdown electric field for devices with various thicknesses. The breakdown electric field for a typical film thickness of 8–15 nm was ∼2–4 MV cm^−1^, which is within observed values for crystalline h-BN^36^. Nevertheless, the deviation occurs. On the basis of our analyses including Raman, TEM, CL and so on, the quality of h-BN is comparable to exfoliated h-BN. Therefore, the defect density might be low. Another source for lower breakdown electric field might be wrinkles. The wrinkles are locally corrugated and may induce the highly localized electric field by the field enhancement factor, resulting in the lower breakdown electric field. We carefully checked AFM data for each device again to clarify the effect of wrinkles. The lower breakdown electric field was observed over the wrinkles (Supplementary Fig. 12).

Utilizing similar devices except a larger 2 × 2 μm^2^ overlap (*A*_device_), the small-signal capacitance of the devices was measured using an Impedance Analyzer (4294A) at 1 kHz modulation frequency. From many devices (*N*=61), *C*_meas_ data as shown in Fig. 4c were fitted with the following equation (1),


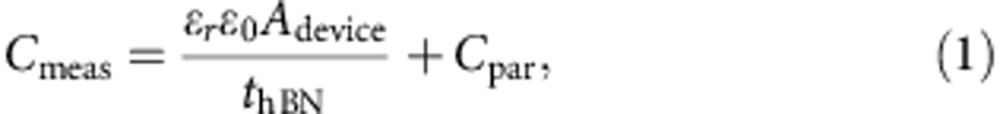


where *ɛ*_*0*_ is the vacuum dielectric permittivity and *C*_par_ is the parasitic capacitance caused by the probing pads. A typical *C*_par_ of ∼15 fF in the relatively thick devices was extracted. The extracted dielectric constant for 61 devices is shown in Fig. 4d. The average relative dielectric constant ranged from 3 to 5, which is consistent with expected values^55^. The large variation of the dielectric constant could be attributed to the effective h-BN thickness due to the presence of wrinkles. The height of wrinkle in a real device is typically ranged from three to six times thicker than that of h-BN film (Supplementary Fig. 13).

### Device applications of multilayer h-BN

**Figure 5:**
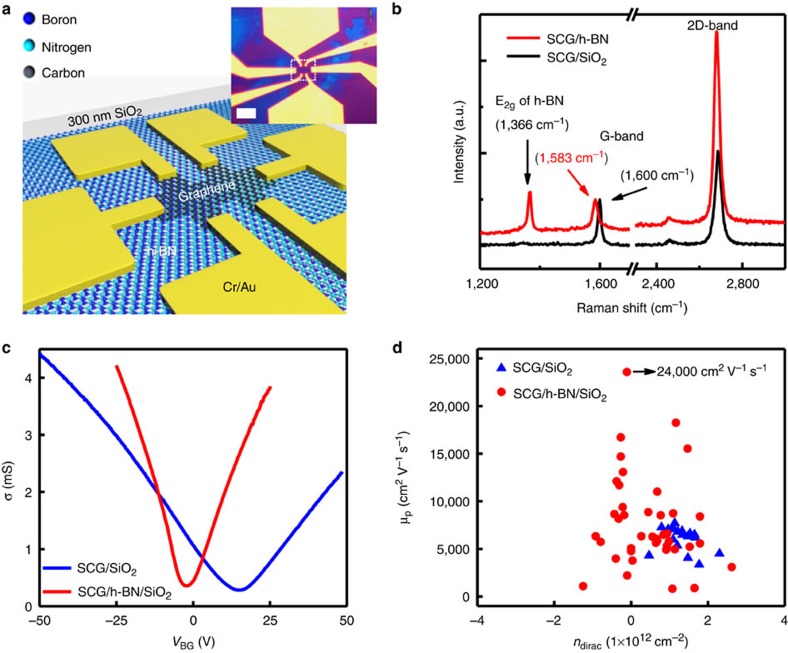
Electrical properties of multilayer h-BN and graphene on an h-BN substrate. (**a**) Schematic diagram of graphene FET on h-BN. The inset shows the optical image of a real device. (**b**) Raman spectra of graphene on a SiO_2_/Si substrate and graphene on a multilayer h-BN substrate. The G-band frequency of graphene on h-BN is near 1,583 cm^−1^, implying that graphene is almost neutral without *p*-doping. (**c**) The representative conductance (*σ*) of the graphene channel as a function of applied back gate (*V*_BG_) at room temperature with a *V*_DS_=100 mV on SiO_2_ (blue) and on h-BN/SiO_2_ (red) substrates. (**d**) The plot of the extracted hole mobility (*μ*_p_) versus *n*_dirac_ for both of the substrates (h-BN and SiO_2_). Scale bar, 10 μm (**a**).

A major application for h-BN is an ideal substrate for 2D materials including graphene and TMdC family. While exfoliated h-BN resulted in very high performance, integration cannot be performed with limited area. Large-area CVD-grown h-BN can be easily integrated with various 2D materials. To demonstrate our h-BN film as a potential substrate, graphene, MoS_2_ and WSe_2_ devices were fabricated on-top of our large-area CVD-grown h-BN^3^. For the CVD graphene devices, a large-area CVD h-BN film was first transferred onto 300-nm-thick SiO_2_/Si wafers, followed by the transfer of large-area single-crystalline CVD-grown graphene (SCG) on the top of a 15-nm-thick h-BN film (Supplementary Fig. 14). A typical size of a single-crystalline graphene domain is between 100 μm to a few hundred micrometres (Supplementary Fig. 15). As a control reference, the same CVD graphene was also transferred onto a reference SiO_2_ wafer. Multi-terminal devices were then fabricated from each sample to avoid any contact resistance artefacts in the measurements, as illustrated in Fig. 5a, showing boron (dark blue), nitrogen (light blue) and carbon (black). The right inset of Fig. 5a shows the optical image of an actual device. The typical channel width (*W*) and length (*L*) are 1.5 and 2 μm, respectively. Before the electrical characterization, the samples were characterized by Raman spectroscopy to confirm the quality of the graphene, including structural defects and doping level^56^. As shown in Fig. 5b, the characteristic phonon modes of h-BN and graphene, such as E_2g_ (1,366 cm^−1^), G-band (1,583 cm^−1^) and 2D band (2,672 cm^−1^) in the Raman spectra were clearly observed. We confirmed that this trend is similar to Raman spectrum of graphene on exfoliated h-BN (Supplementary Fig. 16). The intensity of the D-band (related to structural defects) is negligible, indicating that the single-crystalline graphene is of high quality. Interestingly, the red shift of the G-band, as well as the lower G/2D intensity ratio of the single-crystalline graphene on the h-BN substrate relative to that on SiO_2_/Si indicates a lower *p*-type doping of the single-crystalline graphene on our h-BN substrates. To verify the lower doping and the electrical carrier mobility (*μ*), the conductance (*σ*) of the graphene channel as a function of applied back gate voltage to the conductive silicon (*V*_BG_) was measured (*T*=300 K, *V*_DS_=100 mV) at room temperature. Representative *σ* versus *V*_BG_ is shown in Fig. 5c. From *σ* versus *V*_BG_, both the field-effect hole and electron mobility (*μ*_p_ and *μ*_n_) and the intrinsic doping level (*n*_dirac_) were extracted. The extracted hole mobility (*μ*_p_) and the intrinsic doing (*n*_dirac_) for both substrates (h-BN and SiO_2_) are plotted in Fig. 5d. A summary of the device performance is listed in Supplementary Table 1. Consistently, the average intrinsic doping level of single-crystalline graphene on h-BN is lower than the reference SiO_2_ sample, which might be attributed to the low number of dangling bonds on h-BN surface. This finding also appears to translate to the mobility measurements of single-crystalline graphene. While there is a large variance of single-crystalline graphene quality, the carrier mobility of devices on h-BN substrates exceed over 10,000 cm^2^ V^−1^ s^−1^, with a peak value of ∼24,000 cm^2^ V^−1^ s^−1^, higher than that (∼13,000 cm^2^ V^−1^ s^−1^) with exfoliated h-BN^57^. The higher mobility of graphene in this work might be attributed to both high h-BN quality comparable to exfoliated h-BN and single-crystalline graphene (Supplementary Table 1; Supplementary Fig. 16). The large distribution in values might be related more to the quality of the graphene transfer rather than the variations in the material quality of the h-BN. We investigated numerous high-performance and low-performance devices and found that large bubbles and non-homogeneity during the transfer appear to smear out the conductance curves, resulting in a lower extracted mobility for graphene (Fig. 5d; Supplementary Fig. 14). With improved transfer techniques in the future, higher and more consistent mobility values should be achieved; however, the current h-BN is indeed already useful as a large-area substrate for graphene electronics.

### Optical and electrical properties of MoS_2_ and WSe_2_ on h-BN

**Figure 6:**
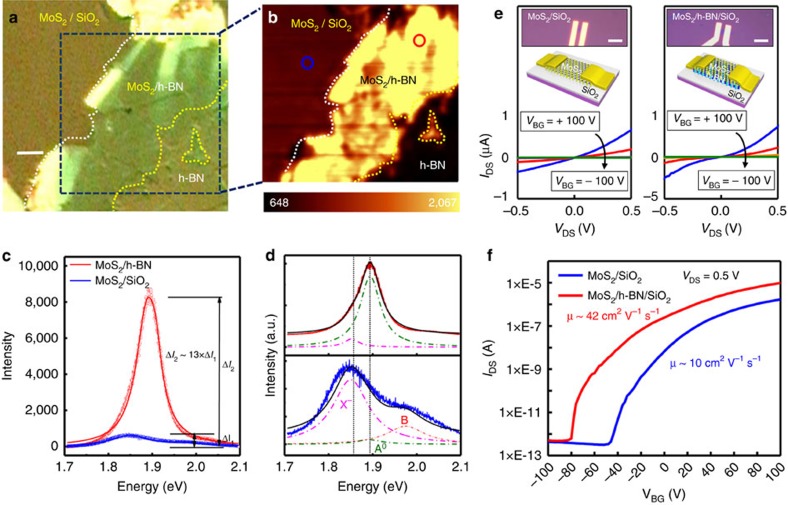
Optical and electrical properties of monolayer MoS_2_ on an h-BN substrate. (**a**) Optical image of monolayer MoS_2_ on a h-BN substrate. The yellow- and white-dashed guidelines indicate the regions of MoS_2_ and h-BN. (**b**) PL mapping image for the exciton emission (near 1.85 eV) for MoS_2_ corresponding to the blue-dashed rectangular region of **a**. (**c**) Representative PL spectra of MoS_2_ on h-BN (red) and SiO_2_ (blue) substrates. The spectra were obtained from red and blue circles in **b**, respectively. (**d**) Lorentzian fitting of the PL spectra for MoS_2_ on h-BN (top) and SiO_2_ (bottom). The PL spectra were fitted with three Lorentzian curves at centres of A° (1.895 eV, neutral exciton), X^−^ (1.852 eV, multiexciton) and B (1.988 eV, neutral exciton). (**e**) *I*_DS_–*V*_DS_ characteristics of monolayer MoS_2_ on SiO_2_ (left) and h-BN (right) substrates for various back gate bias between +100 and −100 V with a step of −20 V. The insets of each panel display the optical image of fabricated FET (top) and corresponsive illustration (bottom). (**f**) Transfer characteristic curves (*I*_DS_–*V*_BG_) of MoS_2_ FET on SiO_2_ and h-BN substrates at *V*_DS_=+0.5 V. Both transfer curves show *n*-type behaviour, but the device on h-BN substrate shows increase in *I*_DS_ and the *n*-type shift of minimum current level towards the negative *V*_BG_. Scale bars, 5 μm (**a**); 10 μm (**e**).

To demonstrate the potential of our h-BN substrate for other 2D semiconducting materials, monolayer MoS_2_ and WSe_2_ on our h-BN substrate were also studied. While graphene does not have a bandgap, 2D TMdCs have an energy bandgap of 1–2 eV (ref. 58), which leads to numerous potential applications such as logic circuits or optoelectronics^2^,^59^,^60^. The optical and electrical properties of TMdCs like those of graphene are also strongly affected by their environmental conditions, especially their substrate^14^. Like graphene, large-area monolayer MoS_2_ and WSe_2_ films were also prepared by CVD (Supplementary Figs 17 and 18). Figure 6 shows the optical properties of monolayer MoS_2_ film on h-BN. Monolayer MoS_2_ film was transferred on h-BN substrate (Fig. 6a). The yellow- and white-dashed guidelines present the regions of MoS_2_ and h-BN, respectively. Since the exciton emission of MoS_2_ strongly depends on the doping level, the sample was further characterized by photoluminescence (PL)^61^. Figure 6b presents the PL mapping image for exciton emission corresponding to the blue-dashed square in Fig. 6a^61^. The exciton emission of MoS_2_ on both h-BN and SiO_2_ is quite uniform in each region, indicating that the monolayer MoS_2_ film was transferred on both substrates uniformly. Interestingly, the exciton emission of MoS_2_ on h-BN is stronger than those on SiO_2_ substrate. Figure 6c displays the representative PL spectra of MoS_2_ on h-BN and SiO_2_ substrate from red and blue circles in Fig. 6b, respectively. The exciton emission of MoS_2_ on a h-BN substrate (Δ*I*_1_) is ∼13 times greater than that of MoS_2_ on a SiO_2_ substrate (Δ*I*_2_), respectively. To understand the origin of the PL intensity of MoS_2_ on a h-BN substrate, the PL spectra were fitted with three Lorentz curves at centres of 1.895 (neutral exciton: A°), 1.852 (multiexciton: X^−^) and 1.988 eV (B neutral exciton; Fig. 6d), respectively^62^. While the weak emissions of both A° and X^−^ on a SiO_2_ substrate in the bottom panel of Fig. 6d were detected, it was found that emission of A° on h-BN substrate is much stronger than that of X^−^ (top), indicating that the neutral exciton emission of MoS_2_ on h-BN is dominant for the PL spectrum. Similar trend for WSe_2_ on a h-BN substrate was observed (Supplementary Fig. 19). This result suggests that both MoS_2_ and WSe_2_ on a h-BN substrate becomes more charge neutral than that on SiO_2_.

We also fabricated a MoS_2_ and WSe_2_ FET device on a h-BN substrate using electron-beam lithography. Electrical properties of palladium (Pd)-contacted MoS_2_ FETs were measured in vacuum. Figure 6e shows the *I*_DS_−*V*_DS_ characteristics of MoS_2_ on SiO_2_ (left) and h-BN (right) substrates for a back gate bias (*V*_BG_) between −100 and +100 V with a step size of 20 V. The insets of each panel display the optical image of the real FET device (top) and the corresponding schematic diagram (bottom). The *I*–*V* curves exhibit nonlinear characteristics on both SiO_2_ and h-BN substrate, indicating that a Schottky barrier was formed at the MoS_2_-Pd contacts. Figure 6f shows the *I*_DS_ of MoS_2_ channel as a function of applied *V*_BG_ to the heavily doped Si at room temperature at a *V*_DS_=0.5 V. The current minimum (*V*_FB_ condition) of MoS_2_ on the SiO_2_ substrate is reached at *V*_BG_=−47 V, showing the *n*-type dominant operation (blue curve). On the other hand, the device on h-BN substrate presents an increase in current level and an *n*-type shift of the minimum current level towards negative *V*_BG_ (red curve) down to −80 V. As a consequence of better contact by reducing the doping level, the mobility of the MoS_2_ FET on the h-BN substrate was enhanced by four times from 10 cm^2^ V^−1^ s^−1^ on SiO_2_ substrate to 42 cm^2^ V^−1^ s^−1^ on h-BN substrate. Similar trend for WSe_2_ FET device was observed (Supplementary Fig. 20). These results prove that our h-BN substrate can be used for enhancing the device performance of 2D materials.

## Discussion

Large-area and high-quality h-BN films were obtained using CVD on a Fe foil with a slow-cooling rate, which allows boron and nitrogen to diffuse out of the iron surface to form multilayer h-BN. More detailed experiments with supporting theoretical calculations would help to elucidate the detailed growth mechanism in the future. The optical, mechanical and electrical properties of h-BN film were characterized using several measurement techniques, indicating that the quality of h-BN is almost similar to single-crystalline h-BN. The CL spectra also proved that the h-BN film can be applied for deep ultraviolet emitter application and for biological sensing applications. In addition, graphene, MoS_2_ and WSe_2_ FET devices on a h-BN substrate were fabricated, resulting in carrier mobilities of graphene, MoS_2_ and WSe_2_ to improve up to ∼24,000, 40 and 9 cm^2^ V^−1^ s^−1^, respectively, indicating the compatibility of our h-BN substrate for 2D electronics. Furthermore, the effect of the h-BN on the doping levels within both the graphene and MoS_2_ (or WSe_2_) transistors suggests that the h-BN can also be used for both substrate/work function engineering of 2D electronics, such as controlling the threshold voltages for complementary logic applications or improving the electrostatics/doping between ohmic interfaces. The large-area and high-quality h-BN substrate in this work not only advances the high performance of 2D nanoelectronics for the future but also provides a new synthesis technique for potential multilayer 2D materials.

## Methods

### Synthesis of multi- and monolayer materials

Multilayer h-BN was synthesized using low-pressure CVD with a borazine precursor. The detailed CVD set-up is described elsewhere^29^. Before synthesis of h-BN, the Fe foil (Alfa Aesar) was annealed at 1,100 °C for 1 h to smoothen the Fe surface under a 10 s.c.c.m. hydrogen atmosphere. For h-BN growth, borazine vapour and hydrogen with a rate of 0.1 and 10 s.c.c.m., respectively, were supplied at 1,100 °C for 30 min. After h-BN growth, different cooling rates (slow: 5 °C min^−1^, fast: 30 °C min^−1^) until 700 °C were applied. For graphene growth, a two-step growth method was applied with low-pressure CVD, which was described elsewhere^63^. Before graphene growth, copper foil (Alfa Aesar) was annealed at 1,040 °C for 30 min under a 10 s.c.c.m. hydrogen atmosphere and then methane and hydrogen with a rate of 0.1 and 10 s.c.c.m., respectively, for 20 min was supplied to reduce the nucleation density, followed by increasing the methane flow rate to 2 s.c.c.m. while maintaining the same flow rate of hydrogen to form a continuous graphene film. To grow monolayer MoS_2_ and WSe_2_ by CVD, MoO_3_ and S for MoS_2_ and WO_3_ and Se for WSe_2_ were used as precursors. The growth of monolayers MoS_2_ and WSe_2_ was conducted at 750 °C for 10 min on a SiO_2_/Si substrate, respectively. The detailed methods were described elsewhere^19^,^20^.

### Transfer of multi- and monolayer materials

To transfer 2D materials onto a target substrate, the conventional poly(methyl methacrylate) (PMMA) transfer method was applied. h-BN/Fe, graphene/Cu and MoS_2_ (or WSe_2_)/SiO_2_ were spin coated with PMMA (C4, Micro Chem) at 2,500 r.p.m. for 1 min and then dried in the oven at 80 °C for 30 min. For a multilayer h-BN film on Fe, the bubbling transfer method was applied^29^. The PMMA/h-BN/Fe was immersed in an aqueous solution of 0.1 M NaOH and used as a cathode. PMMA/h-BN was delaminated from the Fe foil by applying a voltage of 2 V. The PMMA/MoS_2_ (or WSe_2_)/SiO_2_ was immersed in 0.1 M KOH to detach the PMMA/MoS_2_ (or WSe_2_) film from the SiO_2_ substrate. For graphene, the conventional wet-etching transfer was used. Cu was etched by a copper etchant. After all PMMA/h-BN, PMMA/graphene and PMMA/MoS_2_ (or WSe_2_) films were rinsed with distilled water three times, the films were transferred onto the target substrate. The PMMA on 2D materials was removed by acetone and thermal annealing under forming gas at 450 °C. For a graphene, MoS_2_ or WSe_2_ FET device, the h-BN film was first transferred, followed by transferring graphene, MoS_2_ or WSe_2_ on the h-BN substrate. For the cross-sectional TEM measurements, the sample was prepared using a focus ion beam (SMI3050TB, SII).

### Device fabrication and analysis

First, non-oxidizing metal electrodes (10 nm Ti/20 nm Pt) were patterned by lift-off using electron-beam lithography onto a 1-μm thick thermally grown silicon dioxide wafer. Then CVD h-BN was transferred on-top as the gate dielectric following a standard transfer procedure and the second metal electrode (50 nm Pd) was deposited on-top forming a cross-bar structure. 50 nm of Pd was chosen as the top electrode as a low-stress metal to avoid delamination of the underlying h-BN. Furthermore, a thick (1 μm) thermally grown SiO_2_ wafer was chosen to help minimize the parasitic capacitance between the pads and the silicon substrate underneath. For the graphene FET device, large single domains of graphene were transferred onto CVD h-BN/300 nm SiO_2_/Si and 300 an SiO_2_/Si substrates. Ohmic contacts (electron beam evaporated—1 nm Ti/30 nm Au) were patterned first using e-beam lithography and lift-off. Electrical isolation was achieved using PMMA as a etch mask utilizing a reactive oxygen etching tool (Plasma-therm—50 W for 10 s). For the MoS_2_ FET, after the MoS_2_ film was transferred to the h-BN/SiO_2_/Si substrate, the channel and electrode regime were defined using electron-beam lithography, plasma etching (reactive ion etching (RIE): SF_6_ 10 s.c.c.m., 20 W, 10 s), vacuum metallization (Pd/Au, 10/30 nm and a lift-off process, to fabricate two-terminal FET. The devices were annealed at 150 °C for 2 h in 10^−6^ torr to improve the contact between the electrodes and MoS_2_, and also to remove any oxygen-related functional groups on the MoS_2_ surface. The method for the fabrication of WSe_2_ FET device is identical to that of MoS_2_ FET device. For the capacitance measurement, the small-signal capacitance (*C*_meas_) as a function of input frequency (100 Hz to 100 kHz) was recorded utilizing a 50-mV input signal with zero d.c. applied bias, and measured with an Agilent Impedance Analyzer (4294A). For most devices, the capacitances were found to be relatively constant as a function of input frequency. Therefore, *C*_meas_ at a fixed input frequency 1 kHz was measured. Devices were measured at room temperature under vacuum in a Lakeshore VPX Cryoprobe station utilizing an Agilent Semiconductor Parameter Analyzer (4155C). The capacitance was measured using an Agilent Impedance Analyzer (4294A).

### Mobility extraction

Both the field-effect hole and electron mobility (*μ*_p_ and *μ*_n_) and intrinsic doping (*n*_dirac_) were extracted from the maximum slope in Fig. 5c following equation 2:


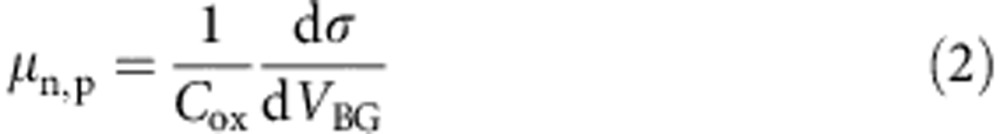


In addition, the back gate charge neutrality point (*V*_CNP_) or Dirac point for the device was extracted and was converted it into a charge-carrier concentration (*n*_dirac_*=C*_ox_/*q* × *V*_CNP_) as a measurement of the intrinsic doping of the samples, where *C*_ox_ is the capacitance of 300 nm SiO_2_/Si.

### Characterization

The surface morphology of the h-BN film was characterized using the AFM (Dimension 3,100 Atomic Force Microscope, Veeco), SEM (Nova NanoSEM 450, FEI) and optical microscopy (Eclipse LV150, Nikon). The quality of h-BN film was characterized using Raman spectroscopy (inVia Raman microscope, Renishaw, 514 nm), X-ray diffraction (SmartLab, Rigaku) and CL (Attolight cathodoluminescence SEM at 10–15 K). The contact angle was measured using a water contact measurement (PHX300, Surface Electro Optics). For mechanical characterizations, an E-sweep (Environment Control Unit) manufactured by Seiko Instruments and diamond-coated AFM tips (Tpa300DLC, Budget Sensors) provided by Budget Sensors were used. To obtain accurate results, the stiffness of the AFM cantilever using the reference cantilever method was determined^64^. The stiffness of the reference cantilever and of the calibrated cantilever was 7.09 and 54.6e s^−1^, respectively. For the structure and elemental analyses, a TEM (Tecnai G2, FEI, 200 kV) and ADF-STEM (ARM200F, JEM) equipped with EELS and selective area diffraction pattern and X-ray photoemission spectroscopy (K-alpha, Thermo scientific) were employed.

## Author contribution

S.M.K. carried out most of the experiments. A.H. and S.H.C. contributed to the device fabrication and analysis. W.F. and J.S.L. contributed to characterize the optical and physical properties of h-BN film. M.H.P. contributed to the characterization of the samples by ADF-STEM. T.P. and M.D. provided advice on the experiments. S.M.K., A.H., K.K.K., Y.H.L. and J.K. designed the experiment. D.-H.C. and C.L. carried out the measurement of mechanical strength. S.J.Y. synthesized the monolayer MoS_2_ and WSe_2_ sample. All the authors discussed the results and contributed to write the manuscript.

## Additional information

**How to cite this article:** Kim, S. M. *et al*. Synthesis of large-area multilayer hexagonal boron nitride for high material performance. *Nat. Commun.* 6:8662 doi: 10.1038/ncomms9662 (2015).

## Supplementary Material

Supplementary Information Supplementary Figures 1-20, Supplementary Table 1 and Supplementary References.
