# Supplementary material for: Synthesis of large-area multilayer hexagonal boron nitride for high material performance
Source: Nat Commun. 2015 Oct 28;6:8662. doi: 10.1038/ncomms9662 (PMC4639899; doi:10.1038/ncomms9662)
Supplement: Supplementary Information — Supplementary Figures 1-20, Supplementary Table 1 and Supplementary References. [file ncomms9662-s1.pdf]

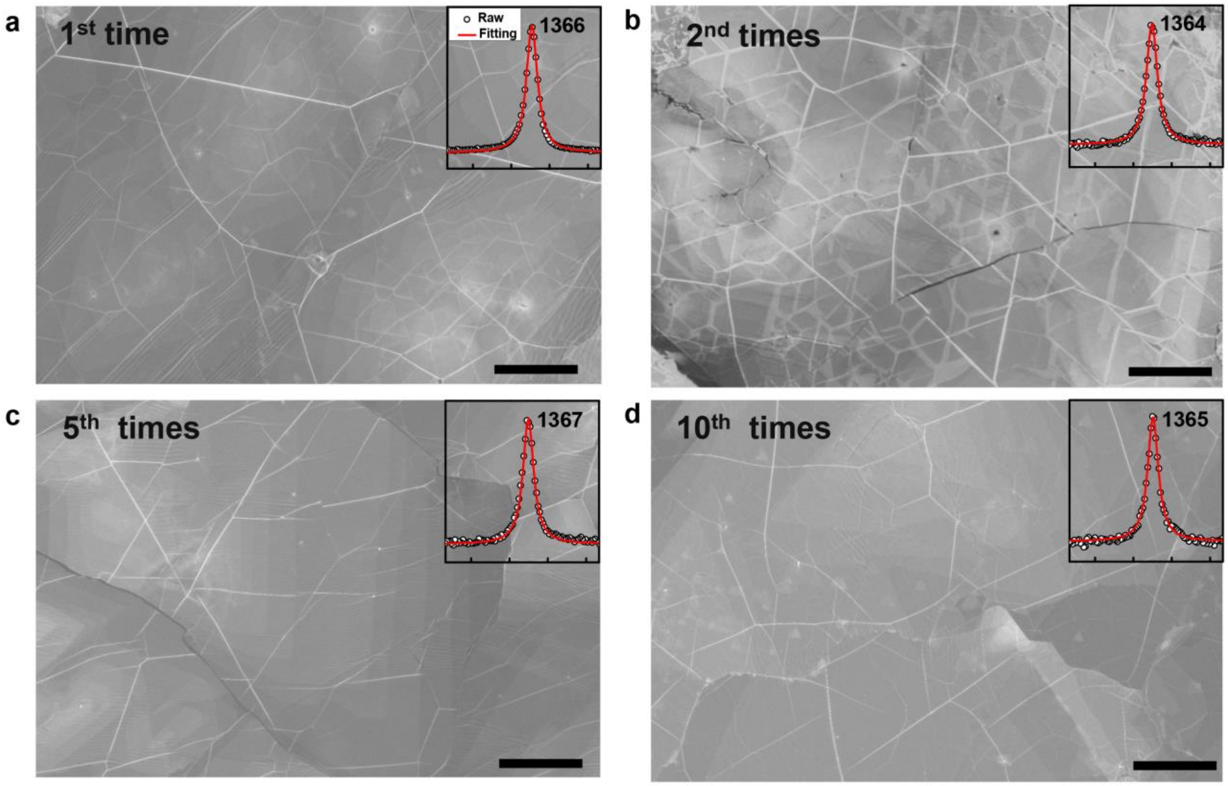

Supplementary Figure 1. (a-d). SEM images of h-BN film on iron foil with corresponding Raman spectra. Iron foil was reused for re-growth of h-BN after bubbling transfer. Scale bars (a-d) 20 μm.

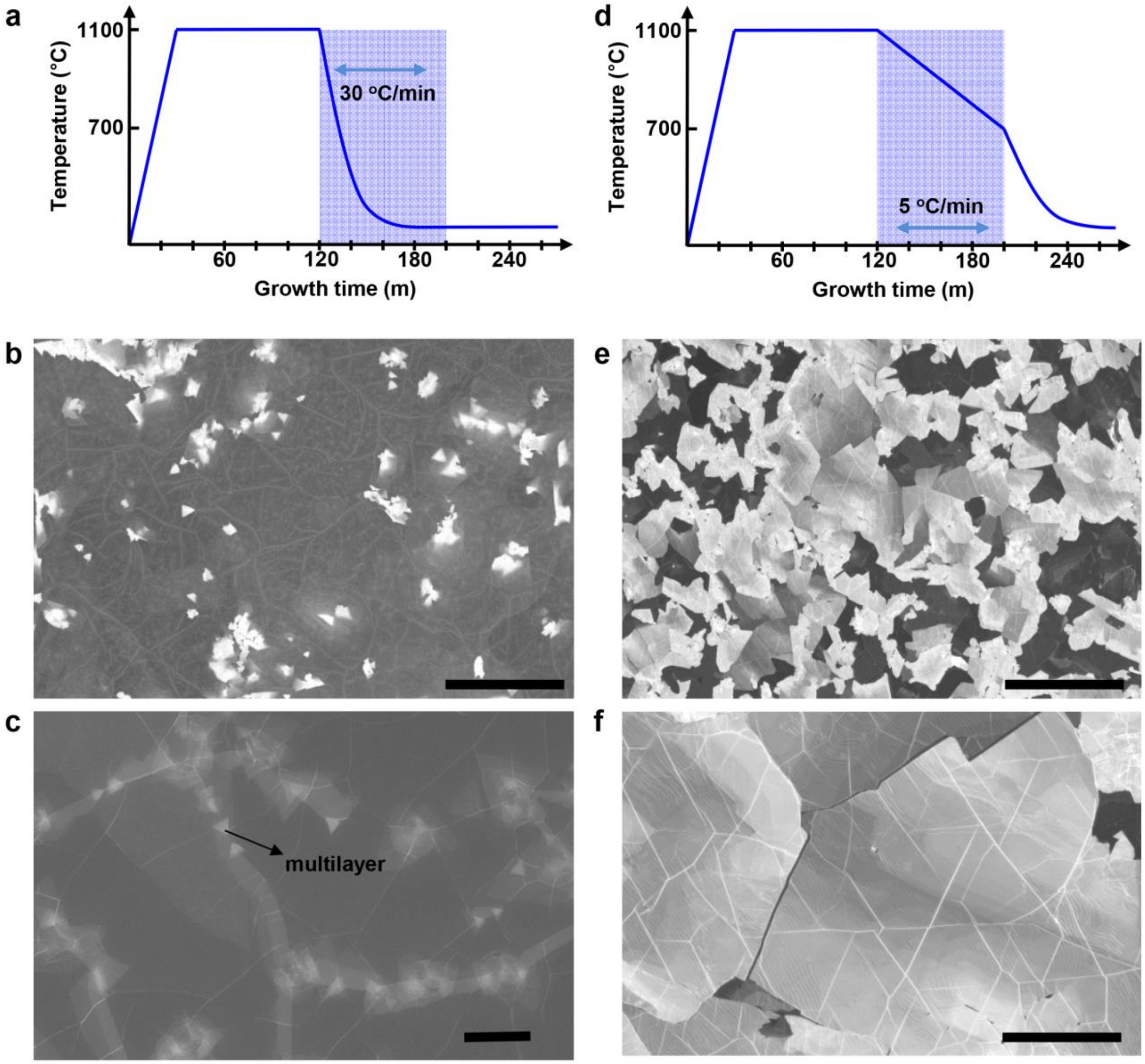

Supplementary Figure 2. Temperature profile of (a) fast cooling and (d) slow cooling rates. SEM images of the resulting h-BN grown via (b-c) fast cooling and (e-f) slow cooling rates. Scale bars (b) 100 μm, (c) 1 μm, (e) 100 μm, (f) 20 μm.

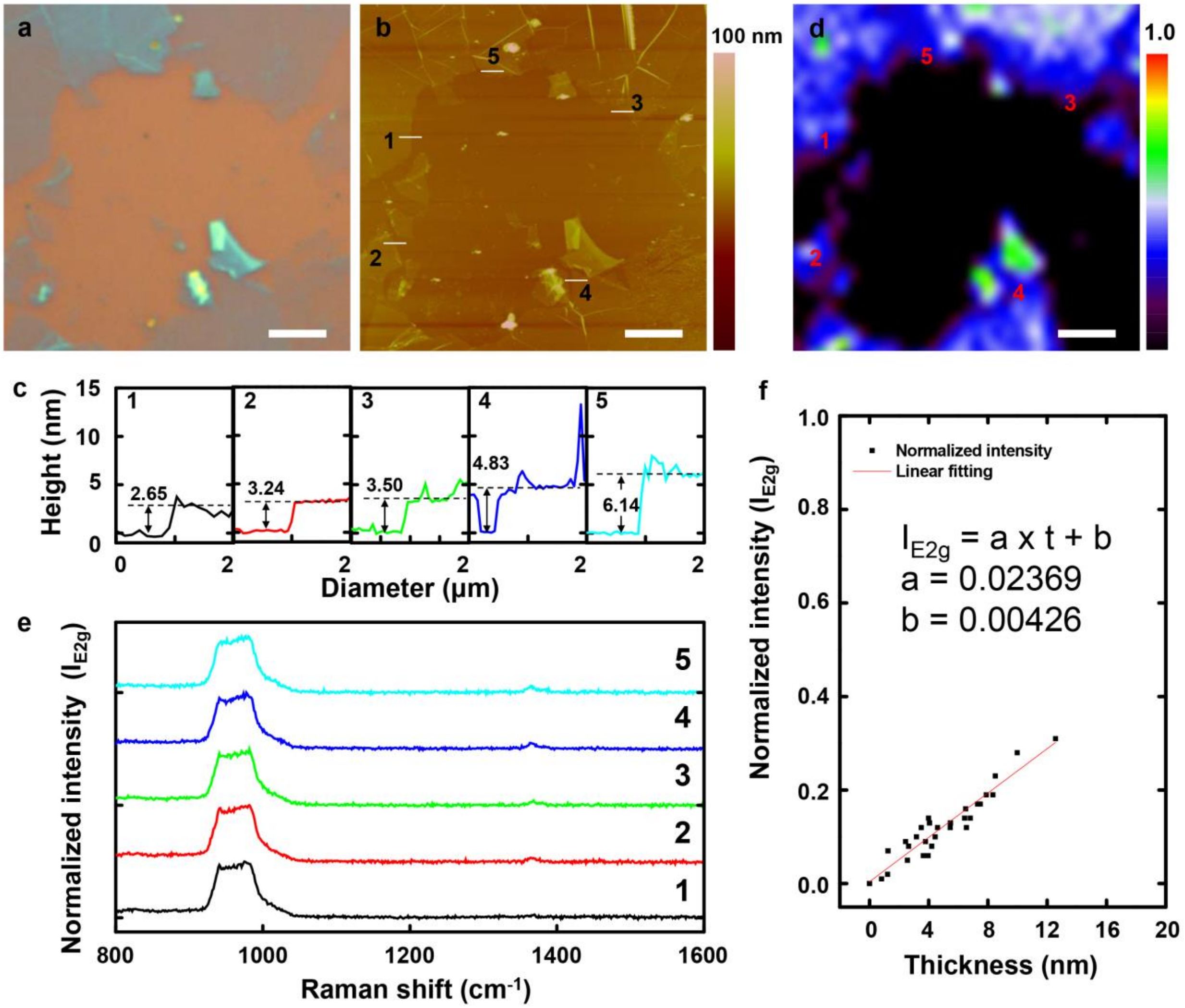

Supplementary Figure 3. (a) and (b) Optical and AFM image of CVD h-BN sample. (c) Thickness of h-BN corresponding to the numbered region in (b). (d) Raman mapping image for the intensity of E_{2g} peak (near 1366 cm^{-1}) of h-BN in the region of (b). Each Raman spectrum was normalized by intensity at 980 cm^{-1} (2TO of Si) to exclude the effect of defocusing during the measurement[1]. (e) Representative Raman spectra of numbered region in (d). Each number in (b-e) presents the same region. (f) Plot of the intensity of E_{2g} (I_{E2g}) as a function of h-BN thickness from 30 data points. It has been reported that I_{E2g} is proportional to thickness of h-BN[2]. To obtain the correlation between I_{E2g} and thickness of h-BN (t), I_{E2g} was fitted with linear plot: I_{E2g} = a x t + b, where a and b are 0.02369 and 0.00426. Based on the linear fitting model, we simply extract the thickness information from the Raman data. Scale bars (a, b, d) 5 μm.

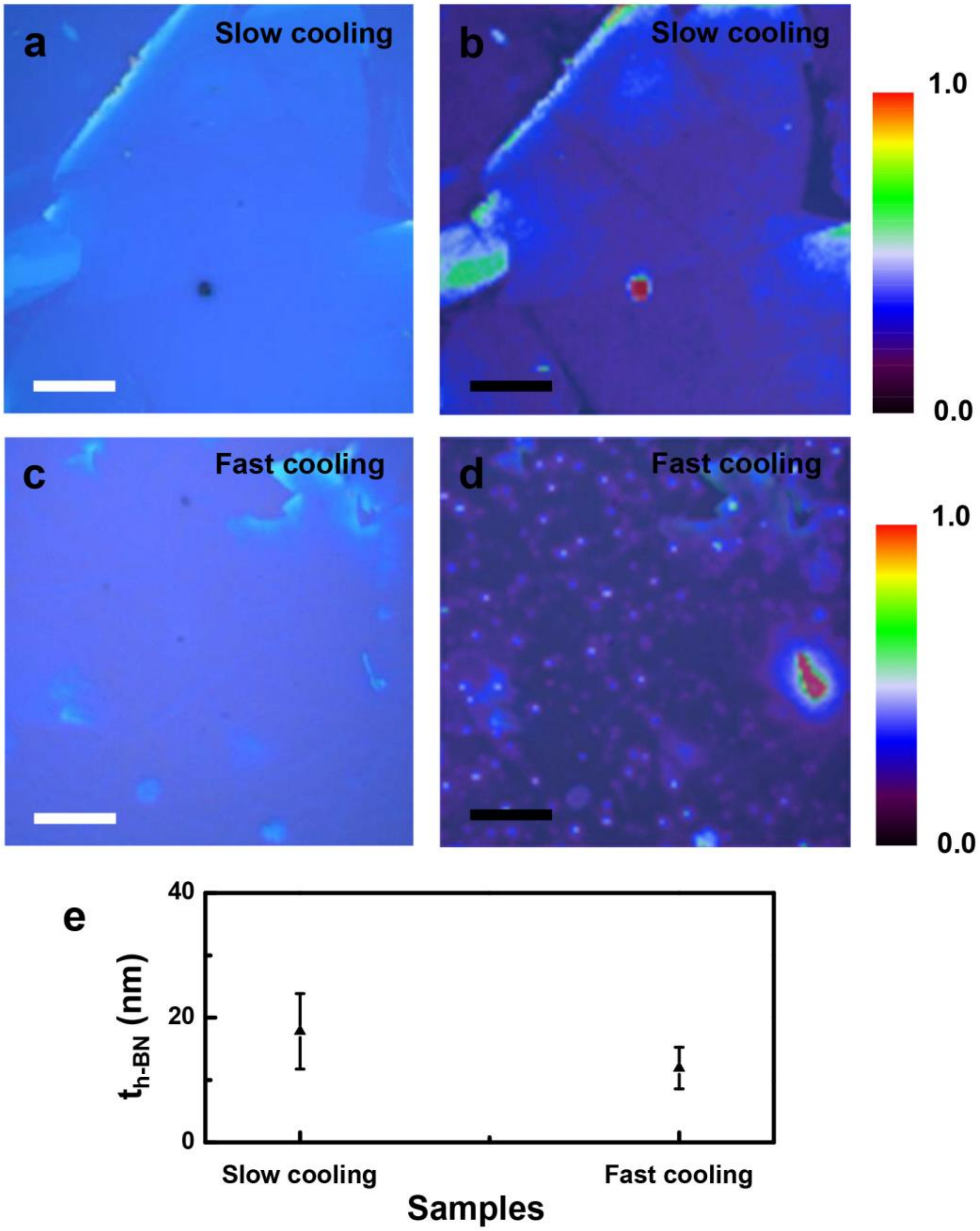

Supplementary Figure 4. Representative optical and corresponding Raman mapping images of I_{E2g} intensity for (a-b) slow and (c-d) fast cooling samples, respectively. (e) Average thickness of slow and fast cooling samples. The thickness of h-BN was deduced by using the linear fitting model: I_{E2g} = a x t + b, where a and b are 0.02369 and 0.00426 from Supplementary Fig. 3. By using the statistics of I_{E2g} from Raman mapping images, the average thickness (s.d.) of slow and fast cooling samples were obtained to be 17.8 nm (6.1 nm) and 11.9 nm (3.3 nm), respectively. Scale bars (a-d) 20 μm.

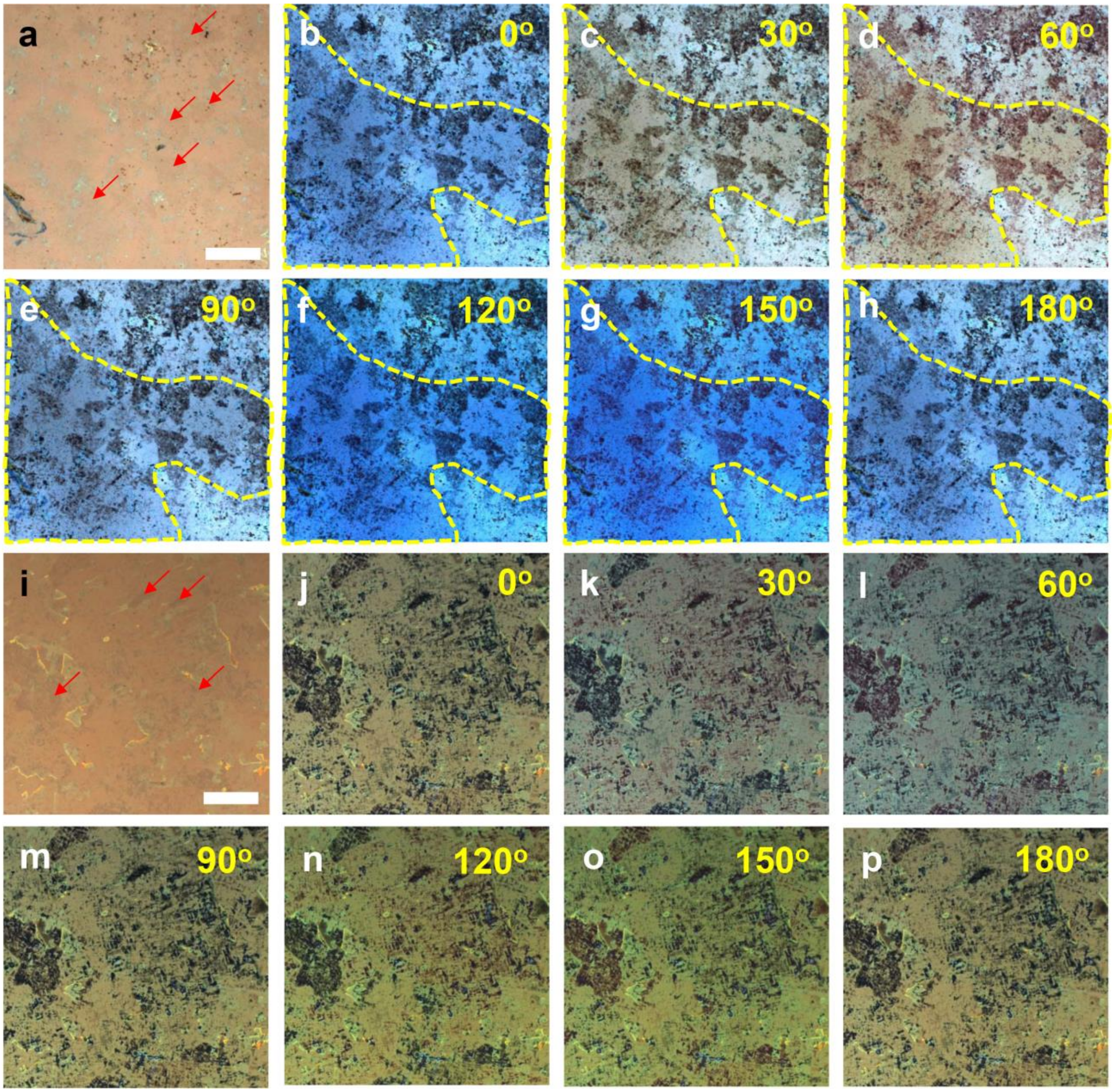

Supplementary Figure 5. (a-h) and (i-p) Optical and corresponding polarizing optical microscopy (POM) images as a function of rotational angles with a step of 30° from 0° to 180° for fast and slow cooling samples, respectively. The optical images of a and i were taken after coating nematic 5CB liquid crystal (LC) on h-BN film/SiO_2/Si. The red arrows in a and i indicate the empty region created by a transfer process. The yellow-dashed lines in b-h guide the different color contrast. It has been reported that the grain in h-BN film can be visualized by the self-alignment of LC along the h-BN grain orientation anisotropically via π-π stacking between h-BN and nematic LC[3]. It is observed that the color in POM images was changed depending on the

rotational angles except the empty regions (black color), where LC was not aligned due to the absence of h-BN. Main difference between fast- and slow-cooled samples is the color contrast in each POM image. While the color contrast for fast-cooled sample was not uniform, guided by dotted-yellow line in b-h, it is quite uniform for slow-cooled sample in j-p. These results indicate that multi-layer h-BN grain in slow cooling sample is aligned over the whole region in the optical image, whereas some h-BN grains in fast cooling sample is misoriented. Therefore, it is estimated that the grain size of h-BN for fast and slow cooling samples were at least 40,000 and 160,000 μm^2, respectively. It is noted that some speckles within the domains were independent of the polarization angle. Scale bars (a, i) 100 μm.

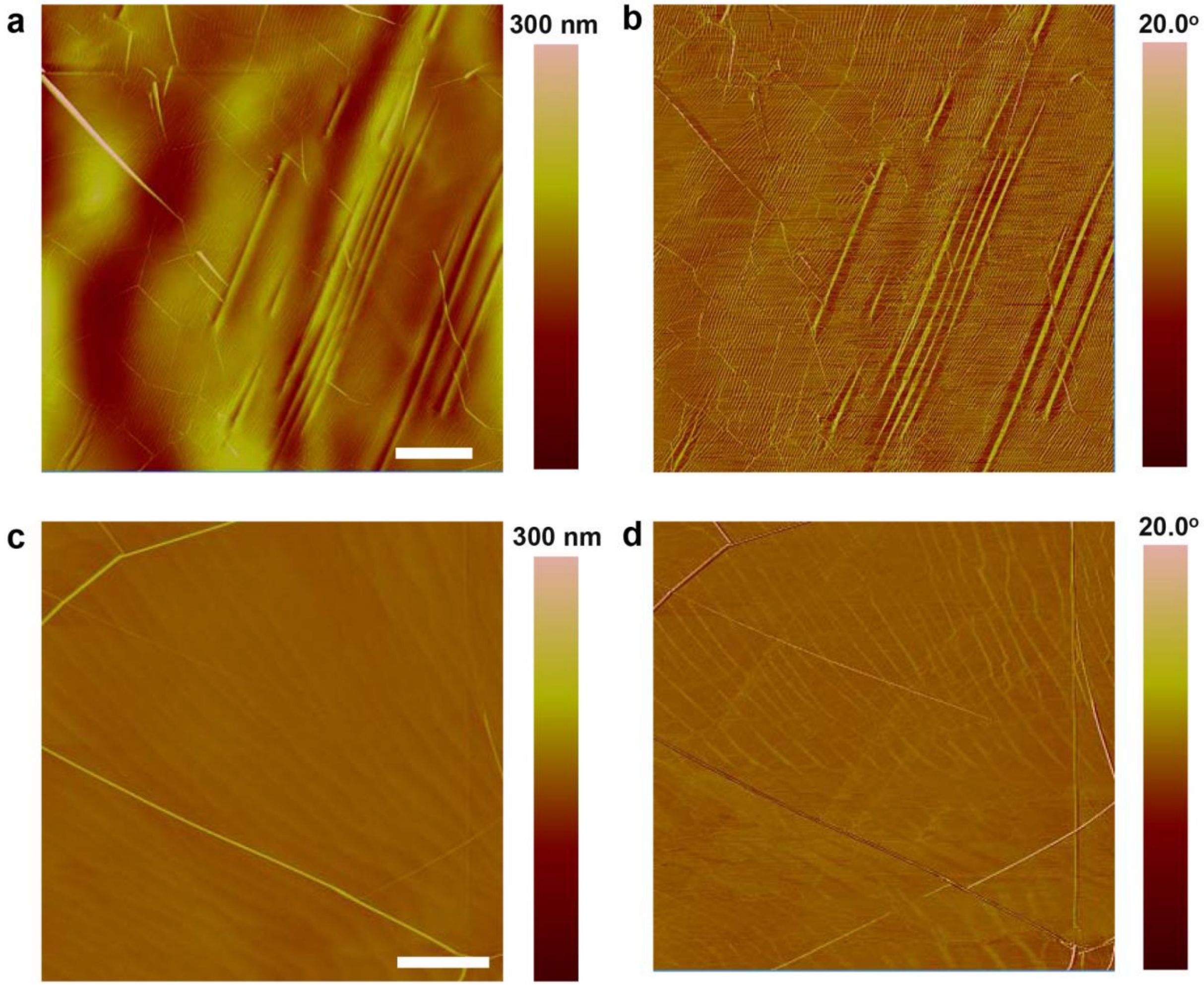

Supplementary Figure 6. (a,c) AFM height and (b,d) phase images of multilayer h-BN on iron foil. The surface of h-BN is quite clean without any impurities. Scale bars (a) 10 μm, (c) 2 μm.

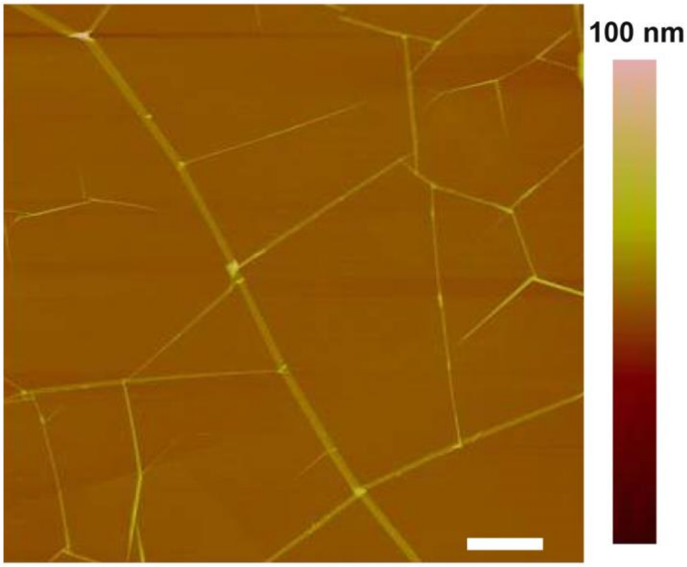

Supplementary Figure 7. AFM image of a multi-layer h-BN on SiO_2/Si substrate after being transferred onto a SiO_2/Si substrate. Scale bar is 2 μm.

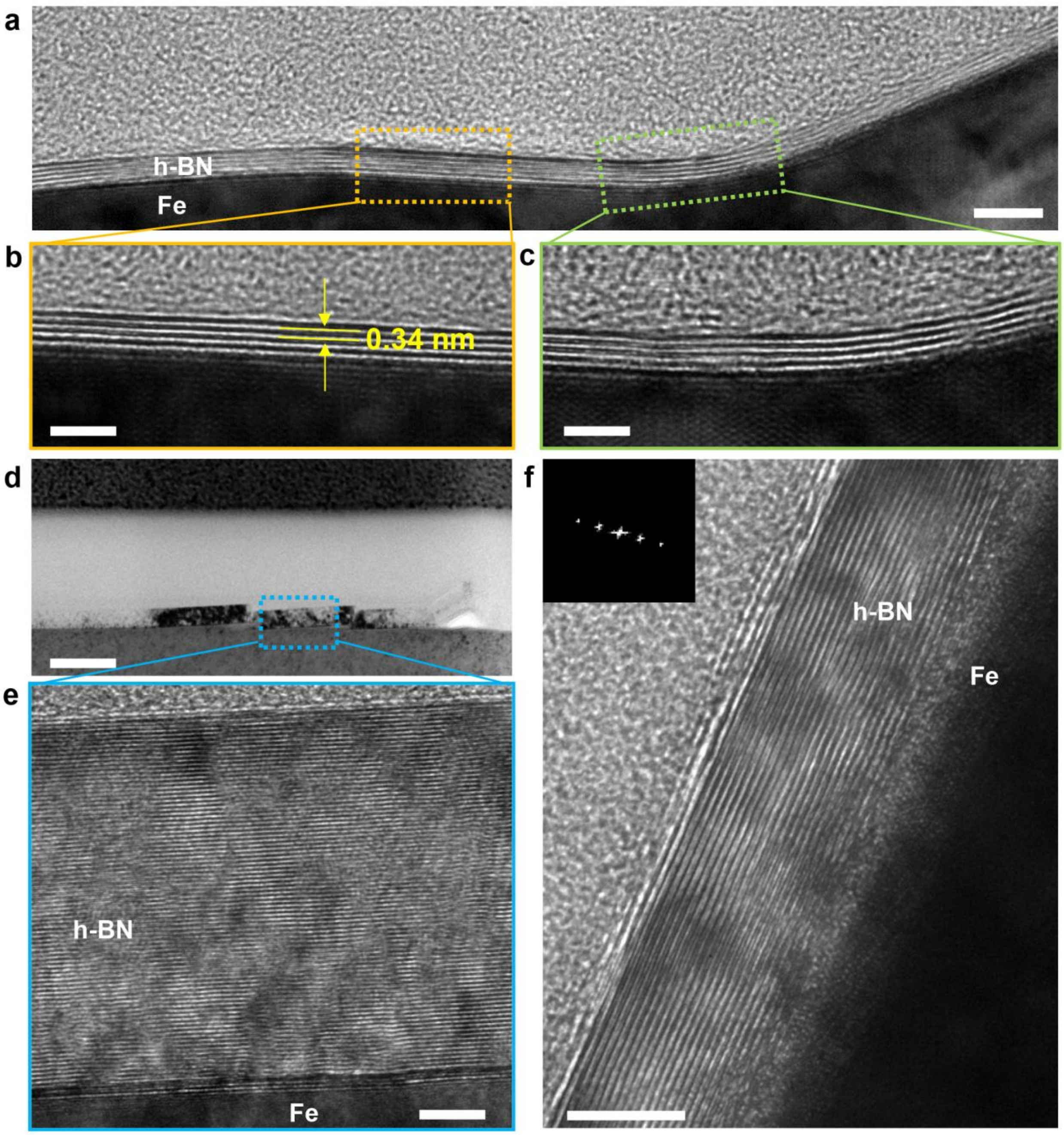

Supplementary Figure 8. (a-f) Cross-sectional TEM images of multilayer h-BN film on iron foil from different regions. The high quality of multi-layer h-BN film was grown. Scale bars (a) 5 nm, (b, c) 3 nm, (d) 100 nm, (e, f) 5 nm.

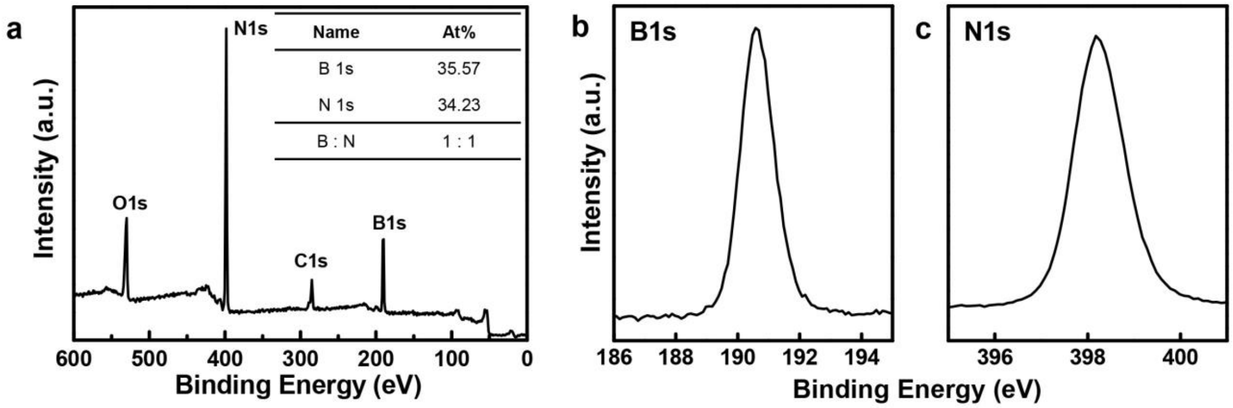

Supplementary Figure 9. XPS spectra of the as-grown multilayer h-BN film on Fe. (a) Wide spectra, (b) and (c) Core level spectra of boron and nitrogen, respectively. The stoichiometry ratio of boron and nitrogen is obtained to be 1:1.

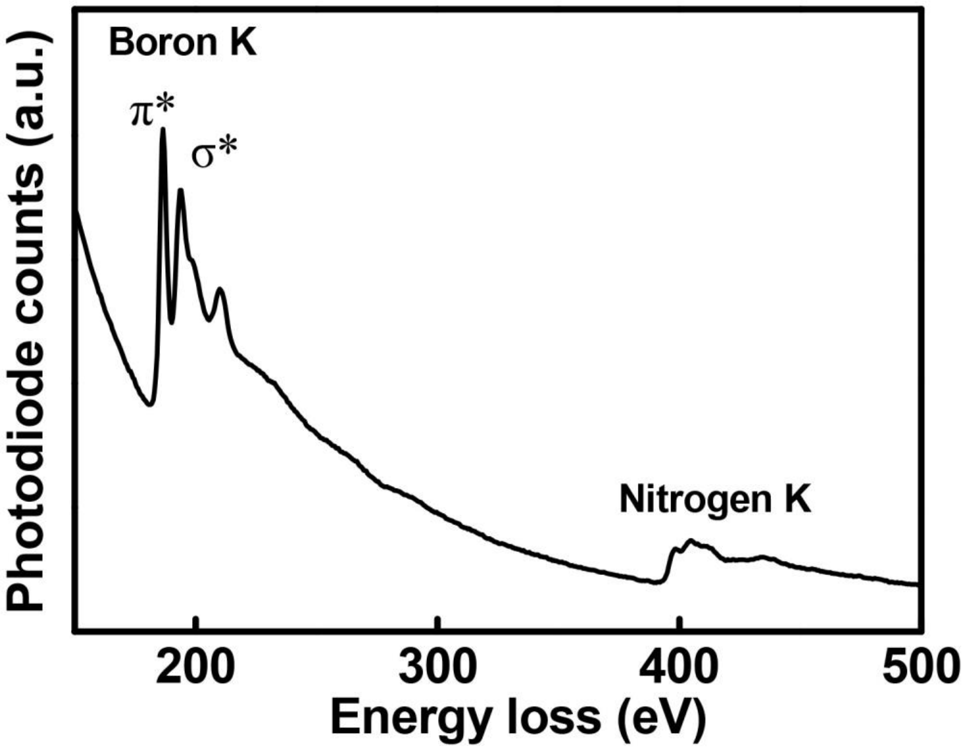

Supplementary Figure 10. The core-loss spectra of the multilayer h-BN film from EELS measurement. Two distinct visible peaks of 180 and 390 eV, corresponding to the characteristic K-shell edges of B and N, respectively. Both π* and σ* energy-loss peaks are present, indicating that the multi-layer h-BN film has sp^2 hybridization bonding[4].

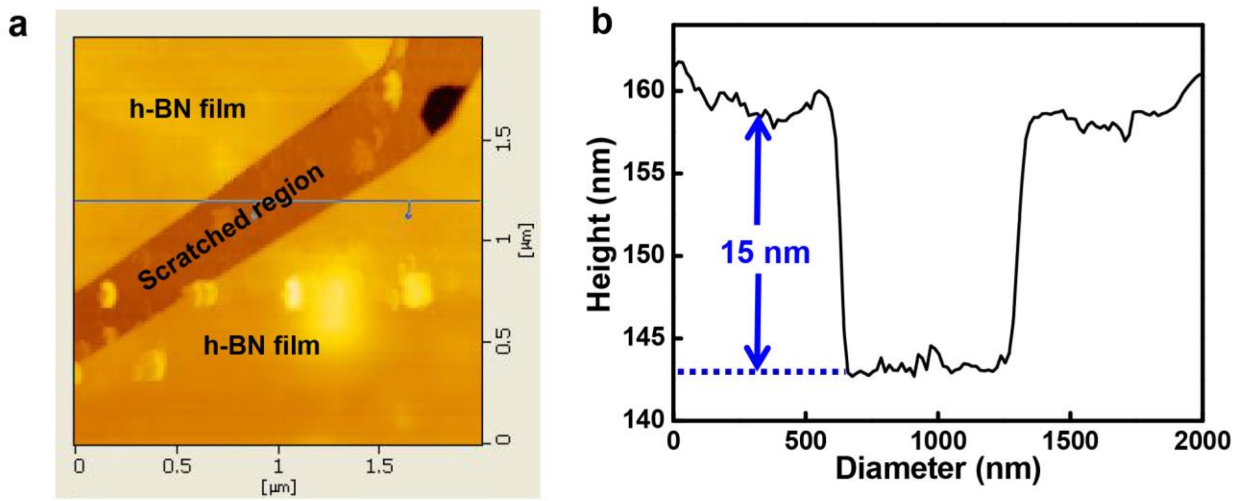

Supplementary Figure 11. (a) AFM image of scratched region for the estimation of h-BN thickness. (b) Height profile along the solid line in (a). The thickness of h-BN for mechanical strength measurement is 15 nm.

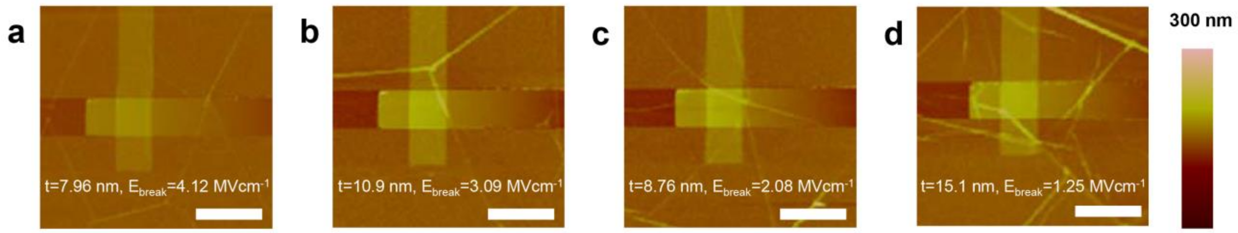

Supplementary Figure 12. (a-d) Representative AFM images of each device for breakdown electric field measurement. Each caption in the image presents the thickness (t) of h-BN and breakdown electric field (E_{break}) of h-BN film. The scale bar is 2 μm. The breakdown electric field without a wrinkle (a) was 4.12 $MVcm^{-1}$, while that value was reduced to 1~3 $MVcm^{-1}$ in the presence of wrinkles (b-d). The wrinkles are locally corrugated and may induce the highly localized electric field by the field enhancement factor, resulting in the lower breakdown electric field. Scale bars (a-d) 2 μm.

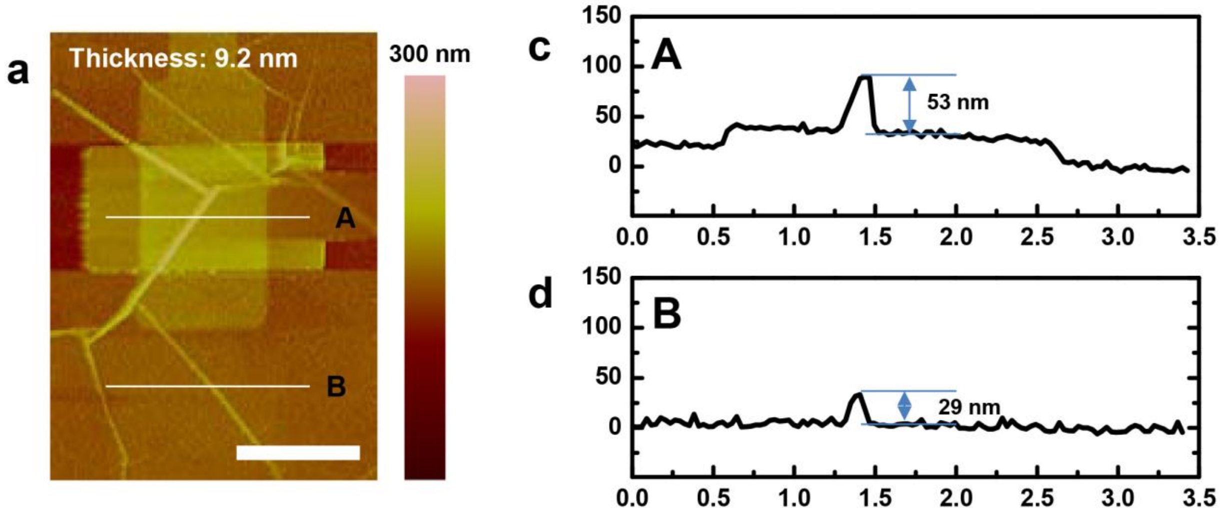

Supplementary Figure 13. (a) AFM image of a real device. (b) and (c) Height profiles along A and B in (a). The thickness of h-BN film is 9.2 nm, which was measured by making trenches around the device and measuring the height profile by AFM. The height of wrinkle is ranged from 3 to 6 times thicker than that of h-BN film. The large variation of capacitance might be related to the variation of effective thickness by the presence of wrinkles. The capacitance is $\varepsilon A_{area}/t_{h\text{-}BN}$, where ε, A_{area} and $t_{h\text{-}BN}$ are dielectric constant, area and thickness of h-BN film, respectively. As effective $t_{h\text{-}BN}$ is increased by the protrusion of wrinkle, the capacitance would be decreased. Scale bar (a) 2 μm.

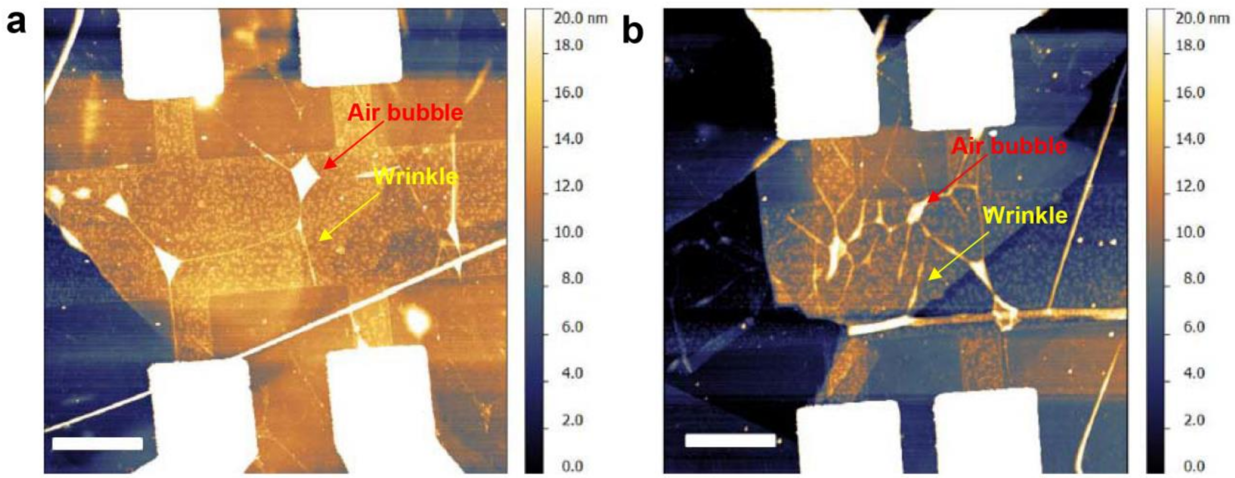

Supplementary Figure 14. AFM images of a (a) high carrier mobility (>10,000 $cm^2V^{-1}s^{-1}$) and (b) low carrier mobility (<2500 $cm^2V^{-1}s^{-1}$) device. The wrinkles and air bubbles of graphene or h-BN are seen, resulting in different conductance of devices. Scale bars (a,b) 1 μm.

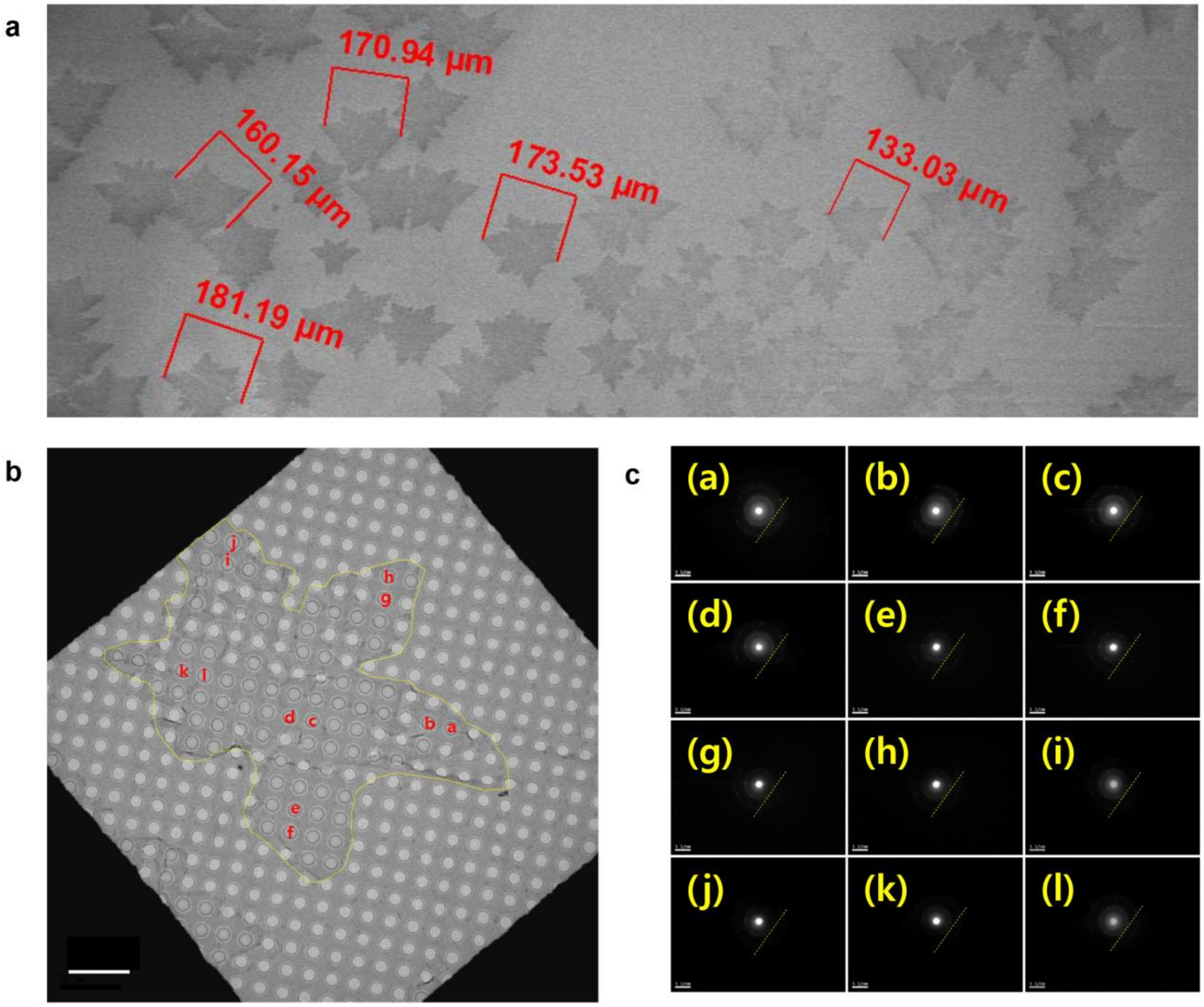

Supplementary Figure 15. (a) SEM image of single crystalline graphene flakes. (b) TEM image of a single crystalline graphene. (c) Selective area diffraction patterns of corresponding to the regions in b. All the hexagonal patterns has same orientation, indicating that graphene is a single crystalline. Scale bar (b) 20 μm.

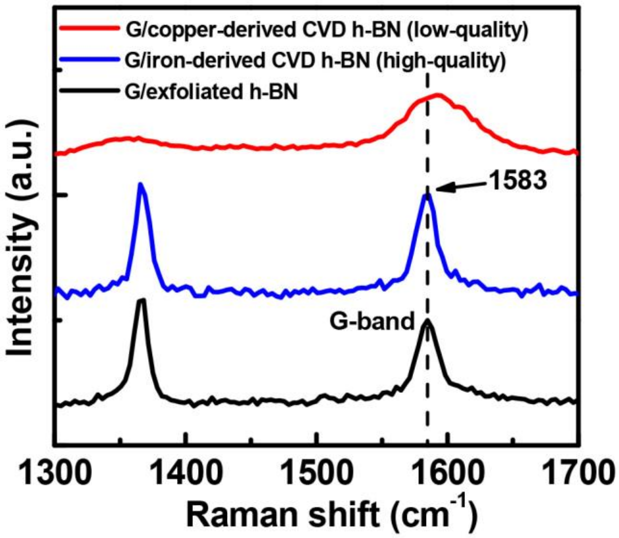

Supplementary Figure 16. Raman spectra of graphene on exfoliated h-BN, iron-derived CVD h-BN (high-quality), and copper-derived CVD h-BN (low-quality)[5] from the top curves. While the G-band of graphene on exfoliated h-BN and iron-derived CVD h-BN substrates is located near 1583 cm^{-1}, Raman spectrum of graphene on copper-derived CVD h-BN shows a different shape including the upshift and broadening of G-band. Low-quality h-BN may have defects on the surface such that they are functionalized by oxygen-related functional groups. This eventually induces doping on graphene, leading to a G-band upshift, which is commonly observed phenomenon[1].

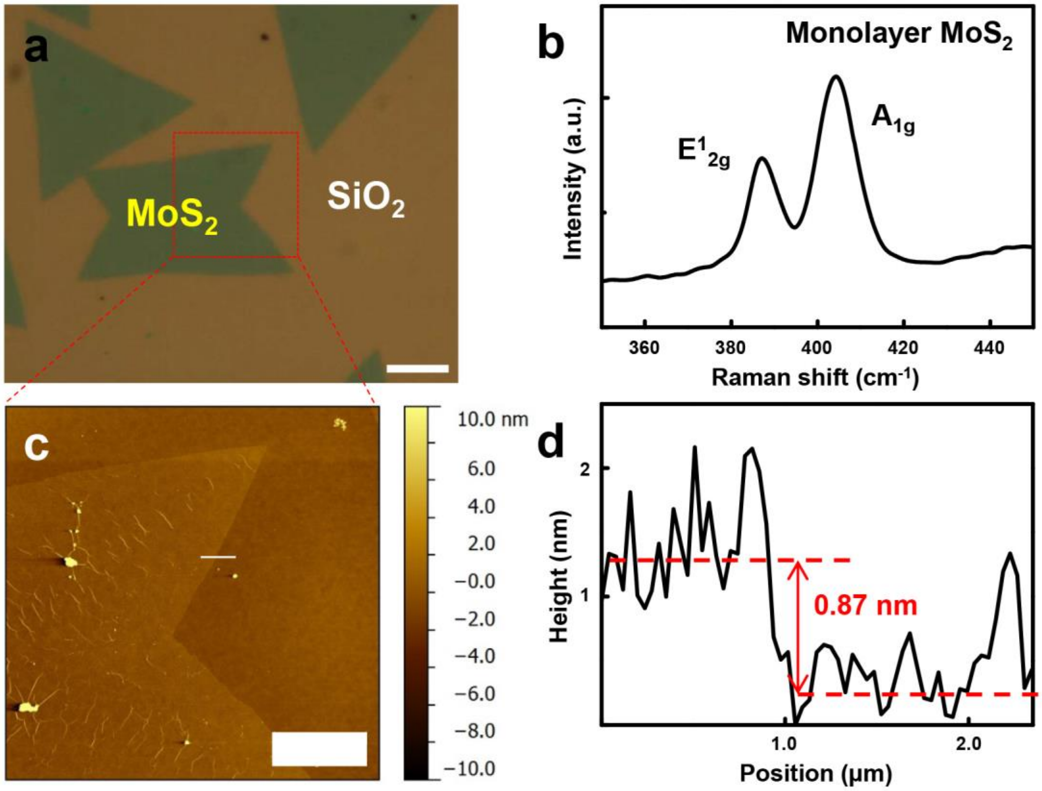

Supplementary Figure 17. (a) Optical image of monolayer MoS_2 film on SiO_2 substrate after transfer. (b) Representative Raman spectrum of monolayer MoS_2. (c) AFM images of monolayer MoS_2 at red-dashed square in (a). (d) Height profile along the white-dashed line in (c). The thickness of monolayer MoS_2 is around 1 nm, indicating that MoS_2 is indeed monolayer. Scale bars (a) 10 μm, (c) 5 μm.

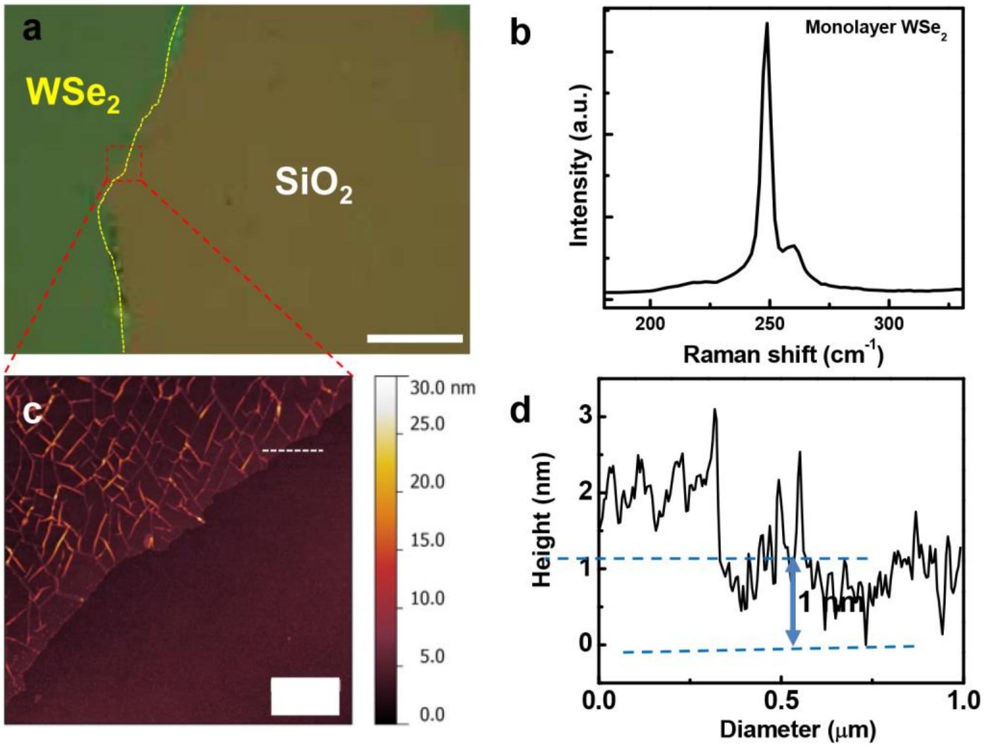

Supplementary Figure 18. (a) Optical image of monolayer WSe_2 film on SiO_2 substrate after transfer. The yellow-dashed line indicates the border between WSe_2 and SiO_2. (b) Representative Raman spectrum of monolayer WSe_2. (c) AFM images of monolayer WSe_2 film at red-dashed square in (a). (d) Height profile along the white-dashed line in (c). The thickness of monolayer WSe_2 film is around 1 nm, indicating that WSe_2 is indeed monolayer. Scale bars (a) 10 μm, (c) 1 μm.

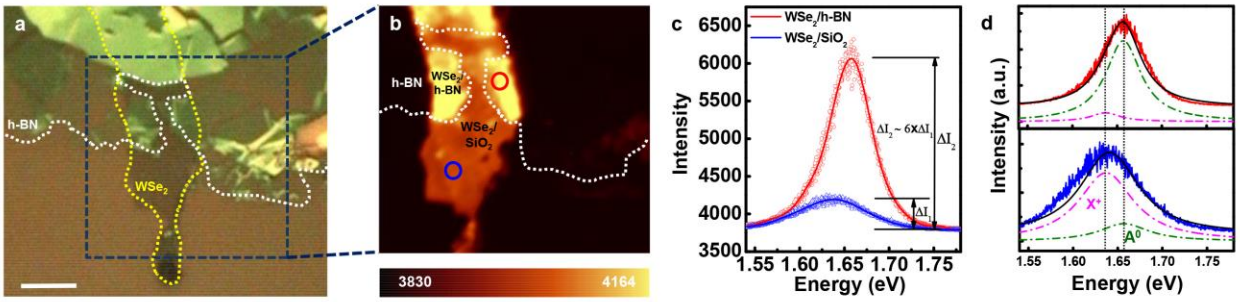

Supplementary Figure 19. (a) Optical image of monolayer WSe_2 on an h-BN substrate. The yellow- and white-dashed guidelines indicate the regions of WSe_2 and h-BN. (b) PL mapping image for the exciton emission (near 1.65 eV) for WSe_2 corresponding to the blue-dashed rectangular region of (a). (c) Representative PL spectra of WSe_2 on h-BN (red) and SiO_2 (blue) substrates. The spectra were obtained from red- and blue-circles in (b), respectively. (d) Lorentzian fitting of the PL spectra for WSe_2 on h-BN (top) and SiO_2 (bottom). The PL spectra were fitted with two Lorentzian curves at centers of A^o (1.657 eV, neutral exciton) and X^+ (1.637 eV, multiexciton)[6, 7]. The exciton emission of WSe_2 on both h-BN and SiO_2 in (b) is quite uniform in each region, indicating that the monolayer WSe_2 film was transferred on both substrates uniformly. The exciton emission of WSe_2 on an h-BN substrate (ΔI_1) is ~ 6 times greater than that of WSe_2 on a SiO_2 substrate (ΔI_2). While the weak emissions of both A^o and X^+ on a SiO_2 substrate in the bottom panel of (d) were detected, that of A^o on h-BN substrate is much stronger than that of X^+ (top), indicating that the neutral exciton emission of WSe_2 on h-BN is dominant for the PL spectrum. This result suggests that WSe_2 on a h-BN substrate becomes more charge-neutral than that on SiO_2. Scale bar (a) 10 μm.

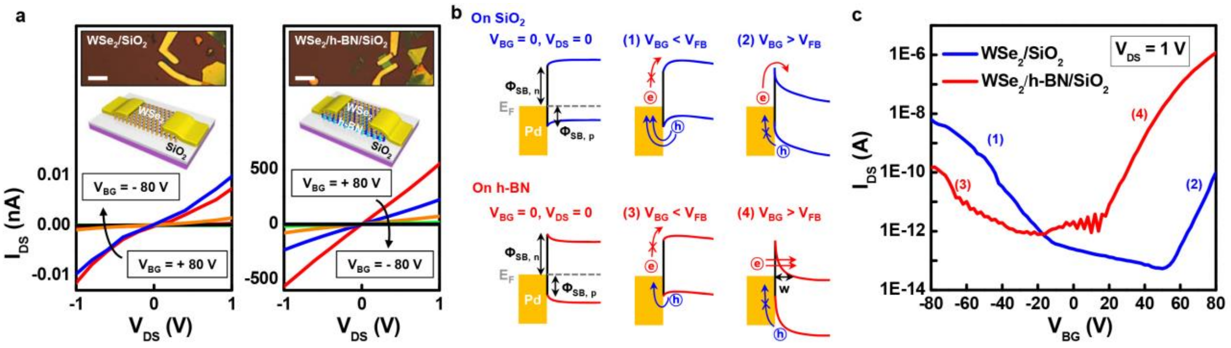

Supplementary Figure 20. (a) I_{DS}-V_{DS} characteristics of monolayer WSe_2 on SiO_2 (left) and h-BN (right) substrates for various back gate bias between + 80 to -80V with a step of -20 V. The insets of each panel display the optical image of fabricated FET (top) and corresponsive illustration (bottom). The I-V curves exhibit nonlinear characteristics on SiO_2 substrate, indicating that a Schottky barrier was formed at the WSe_2-Pd contacts. On the other hand, the I-V curves on the h-BN substrate exhibit more linear dependence, suggesting that ohmic-like contacts were formed between WSe_2 and Pd electrodes. (b) Illustration of band diagram of WSe_2 FET: Band bending without any bias and with gate voltages (V_{GB}) below and above the flat band bias (V_{FB}) conditions at a positive V_{DS} for WSe_2 FET on SiO_2 (blue lines, (1) and (2)) and for WSe_2 FET on h-BN (red lines, (3) and (4)), respectively. Φ_{SB}, w, e, and h are standing for the height of Schottky barrier, depletion width, electron, and hole, respectively. In the case of WSe_2 FET on SiO_2, the Schottky barrier appears in both hole- ($V_{BG} < V_{FB}$) and electron-doping ($V_{BG} > V_{FB}$) bias regime ((1) and (2)). On the other hand, if WSe_2 on our h-BN substrate is less p-doped than that of WSe_2 on the SiO_2/Si substrate, strong band bending at the Pd/WSe_2 interface results in a reduced potential barrier enabling a larger tunneling current at electron doping bias ($V_{BG} > V_{FB}$) regime (4). (c) Transfer characteristic curves (I_{DS}-V_{BG}) of WSe_2 FET on SiO_2 and h-BN substrates at V_{DS}= +1 V. Both transfer curves show ambipolar behavior, but the device on h-BN substrate shows increase in I_{DS} and the n-type-shift of minimum current level toward to negative V_{BG}. As a consequence of better contact by reducing doping, the mobility of WSe_2 FET on h-BN substrate was enhanced by 150 times from 0.06 $cm^2V^{-1}s^{-1}$ on SiO_2 substrate to 9 $cm^2V^{-1}s^{-1}$ on h-BN substrate. Scale bars (a, b) 10 μm.

Supplementary Table 1. Averaged electron and hole mobility of a single crystalline graphene (SCG) and polycrystalline graphene (PCG) on SiO_2 and h-BN from several devices.

Graphene quality	Substrate	Transfer condition	n_{dirac} (10^{12} cm^{-2})	μ_n ($cm^2V^{-1}s^{-1}$)	μ_p ($cm^2V^{-1}s^{-1}$)	Number of devices
PCG*	SiO_2	Annealing	1.68±0.28	3690±1448	3102±1369	30
SCG	SiO_2	Annealing	1.31±0.41	5494±1256	6091±1252	18
SCG	CVD h-BN/SiO_2	Annealing	0.71±1.27	4407±3230	7490±4773	44

*The growth condition for PCG was followed by the previous work[8].

References:

1. Quiroga-González E, Carstensen J, Glynn C, O'Dwyer C, Föll H. Pore size modulation in electrochemically etched macroporous p-type silicon monitored by FFT impedance spectroscopy and Raman scattering. *Phys. Chem. Chem. Phy*. **16**, 255-263 (2014).

2. Gorbachev RV*, et al.* Hunting for monolayer boron nitride: optical and Raman signatures. *Small* **7**, 465-468 (2011).

3. Park J-H*, et al.* Large-Area Monolayer Hexagonal Boron Nitride on Pt Foil. *ACS Nano* **8**, 8520-8528 (2014).

4. Huang JY, Yasuda H, Mori H. HRTEM and EELS studies on the amorphization of hexagonal boron nitride induced by ball milling. *J Am. Ceram. Soc.* **83**, 403-409 (2000).

5. Kim KK*, et al.* Synthesis of Monolayer Hexagonal Boron Nitride on Cu Foil Using Chemical Vapor Deposition. *Nano Lett.* **12**, 161-166 (2012).

6. Jones AM*, et al.* Optical generation of excitonic valley coherence in monolayer WSe2. *Nature Nanotech.* **8**, 634-638 (2013).

7. Tonndorf P*, et al.* Photoluminescence emission and Raman response of monolayer MoS2, MoSe 2, and WSe2. *Optics Express* **21**, 4908-4916 (2013).

8. Wang QH*, et al.* Understanding and controlling the substrate effect on graphene electron-transfer chemistry via reactivity imprint lithography. *Nature Chem.* **4**, 724-732 (2012).
